# Exploring the synergistic effects of cabozantinib and a programmed cell death protein 1 inhibitor in metastatic renal cell carcinoma with machine learning

**DOI:** 10.18632/oncotarget.28183

**Published:** 2022-01-27

**Authors:** Ignacio Durán, Daniel Castellano, Javier Puente, Lidia Martín-Couce, Esther Bello, Urbano Anido, José Manuel Mas, Luis Costa

**Affiliations:** ^1^Medical Oncology Department, University Hospital Marqués de Valdecilla, IDIVAL, Santander, Spain; ^2^Medical Oncology Department, University Hospital 12 de Octubre, Madrid, Spain; ^3^Medical Oncology Department, Hospital Clínico San Carlos, Instituto de Investigación Sanitaria del Hospital Clínico San Carlos (IdISSC), CIBERONC, Madrid, Spain; ^4^IPSEN, Planta 7, Torre Realia, L’hospitalet de Llobregat, Barcelona, Spain; ^5^Department of Medical Oncology, University Clinic Hospital of Santiago, Health Research Institute (IDIS), ONCOMET, Santiago de Compostela, Spain; ^6^Anaxomics Biotech, Barcelona, Spain; ^7^Oncology Department, Hospital de Santa Maria, Centro Hospitalar Universitário Lisboa Norte, Lisbon, Portugal; ^8^Instituto de Medicina Molecular-João Lobo Antunes, Faculdade de Medicina, Universidade de Lisboa, Lisbon, Portugal

**Keywords:** machine learning, cabozantinib, renal cell carcinoma, tumour microenvironment, systems biology

## Abstract

Clinical evidence supports the combination of cabozantinib with an immune checkpoint inhibitor for the treatment of metastatic clear cell renal cell carcinoma (mccRCC) and suggests a synergistic antitumour activity of this combination. Nevertheless, the biological basis of this synergy is not fully characterized. We studied the mechanisms underpinning the potential synergism of cabozantinib combined with a PD1 inhibitor in mccRCC and delved into cabozantinib monotherapy properties supporting its role to partner these combinations. To model physiological drug action, we used a machine learning-based technology known as Therapeutic Performance Mapping Systems, applying two approaches: Artificial Neural Networks and Sampling Methods. We found that the combined therapy was predicted to exert a wide therapeutic action in the tumour and the microenvironment. Cabozantinib may enhance the effects of PD1 inhibitors on immunosurveillance by modulating the innate and adaptive immune system, through the inhibition of VEGF-VEGFR and Gas6-AXL/TYRO3/MER (TAM) axes, while the PD1 inhibitors may boost the antiangiogenic and pro–apoptotic effects of cabozantinib by modulating angiogenesis and T-cell cytotoxicity. Cabozantinib alone was predicted to restore cellular adhesion and hamper tumour proliferation and invasion. These data provide a biological rationale and further support for cabozantinib plus PD1 inhibitor combination and may guide future nonclinical and clinical research.

## INTRODUCTION

Renal cell carcinoma (RCC) accounts for approximately 90% of all kidney cancers [1] and is the seventh most common diagnosed malignancy with an increasing incidence in developed countries [2]. The dominant subtype occurring in 75% of cases is clear cell renal cell carcinoma (ccRCC) [3] and, despite the increasing understanding of cancer biology, fewer than 13% of patients with metastatic ccRCC (mccRCC) survive beyond five years [2, 4, 5]. RCC is characterized by frequent loss–of–function of the von Hippel Lindau (VHL) gene, which makes it one of the most hyper–vascularized tumours [2, 3]. This is related to HIF1α and HIF2α accumulation and overexpression of genes related to hypoxia response such as VEGF (Vascular Endothelial Growth Factor), PDGF (platelet derived growth factor) and others leading to promotion of angiogenesis, tumour growth and survival [2, 3, 6, 7]. An immunosuppressive tumour microenvironment (TME) is another hallmark of RCC, depicted by angiogenic mediators, chemokines, and defective T-cells with dysfunctional cytotoxicity affected by checkpoint regulation and by the myeloid-derived suppressor cells (MDSC) immunosuppressive activity [6].

There have been dramatic changes in the therapeutic landscape since cytokine immunotherapies provided the mainstay of mccRCC treatment in the 1990s [3, 8]. The greater understanding of the molecular biology of mRCC with advances in “omics” technologies gave way to the tyrosine kinase inhibitors (TKI) era in the 2000s, with the development of targeted therapies to hamper the VEGFR and PI3K/AKT/mTOR pathways [3]. Despite the successes, VEGF pathway blockade is associated with drug resistance. Further insights over the past decade on how tumours take advantage of immunosuppressive regulatory mechanisms to evade the immune system led to the development of the immune checkpoint inhibitors (ICI) [3, 8]. Pharmacological disruption of the programmed cell–death protein 1 (PD1) and the cytotoxic T-lymphocyte–associated antigen 4 (CTLA-4) pathways [8] provided significant improvements in survival and quality of life of patients [3, 9]. Clinical data point to a role for these PD1 inhibitors on reactivation of tumour immunosurveillance mechanisms [8, 9]. Also, blockade of PD1 signalling in cytotoxic T-cells was shown to promote their expansion and survival, indirectly contributing to antitumour responses [10]. Currently, anti-PD1 (nivolumab, pembrolizumab), anti-PDL1 (atezolizumab, avelumab, durvalumab) and anti-CTLA-4 (ipilimumab, tremelimumab) monoclonal antibodies (mAbs) represent the ICI landscape for RCC [3]. However, the lack of clinical benefit in a proportion of patients [11] limit their use as monotherapies, which led to their combination with other immunomodulatory or antiangiogenic therapies [9]. Moreover, the appearance of resistances with double ICI combinations together with the immunomodulatory potential of VEGF inhibitors paved the way for the anti-VEGF/VEGFR TKI plus ICI combinations.

Among the TKI inhibitors, cabozantinib has unique antitumour and immunomodulatory properties due to its greater number of targets, supporting its role as suitable partner for checkpoint inhibitors [11, 12]. Thus, apart from impairing VEGF signalling, cabozantinib inhibits a variety of receptor tyrosine kinases including MET and TAM kinases (TYRO3, AXL, MER) involved in tumour growth, metastasis, and therapeutic resistance with a role in immunosuppression [11–13]. Specifically, cabozantinib was shown to decrease tumour and endothelial cell proliferation, increase apoptosis and inhibit tumour growth in breast, lung, and glioma tumour models [13]. On the other hand, several studies have highlighted the antitumour immunomodulatory activity exerted both on immune cells and tumour cells, which promote a tumour immune–permissive environment and enhance tumour vulnerability to immunotherapy [11, 12]. Nonclinical and clinical data have validated the suitability of cabozantinib as a partner for ICI combinations and suggest synergistic antitumour activity in patients with mRCC [11]. In the phase 3 ChecMate9ER study, cabozantinib plus nivolumab demonstrated improved efficacy, while maintaining health-related quality of life, compared with sunitinib in previously untreated patients with advanced RCC [14]. These data have positioned cabozantinib plus nivolumab as front-line therapy in the treatment algorithm for mccRCC along with axitinib plus pembrolizumab, ipilimumab (anti-CTLA-4) plus nivolumab and lenvatinib-pembrolizumab [15, 16].

However, the synergistic mechanisms in the complex cellular TME are unclear and difficult to unravel judged by the intricate interactions among intervening cells —tumour, immune and endothelial cells as well as other cell types of the tumour stroma [7] —and biological entities. In addition, variables like genetic intratumoural heterogeneity [17], inter–individual variability, drug resistance, lack of response or severe toxicities complicate the picture. Thus, uncovering the molecular mechanisms driving combinatory drug actions will be of help to tailor treatments and optimize therapeutic responses.

For this reason, we set out to identify the mechanisms underpinning the potential synergism of cabozantinib combined with a PD1 inhibitor in mccRCC and explore the advantages for its reported efficacy. In addition, we investigated the biological basis for the therapeutic action of cabozantinib considering its complete target profile. To integrate multiple host– and tumour–specific variables at play, we used previously described systems biology– and machine learning –based techniques (Therapeutic Performance Mapping System, TPMS) [18] to model the mechanism of action of frontline treatment cabozantinib plus a PD1 inhibitor in mRCC. To this aim, we used two approaches: Artificial Neural Networks and Sampling Methods [19, 20]. Based on information available in the databases, the first approach provides predictive capacity to evaluate the potential relationship —assumed as therapeutical effect— between the target proteins of a drug and the effector proteins involved in the pathology of interest; the second approach offers descriptive capacity to trace the most probable paths —in biological and mathematical terms— that lead from a stimulus (drug) to a response through the human protein network. The application of bioinformatics and systems biology to generate pathophysiology–feasible models elicits great interest in drug development and regulatory decision fields for its potential to identify molecular–level mechanistic details [21]. Systems–biology methods have aided in untangling the molecular effects of drugs in complex clinical settings [18, 20, 22–24], including cancer [19]. This novel in silico approach, not previously used in the field of renal cancer, entails a holistic manner to give response to complex (patho)physiological drug–related questions still unresolved by clinical trials.

## RESULTS

### Mechanistic models of cabozantinib and a PD1 inhibitor in metastatic clear cell renal cell carcinoma

**Figure 1:**
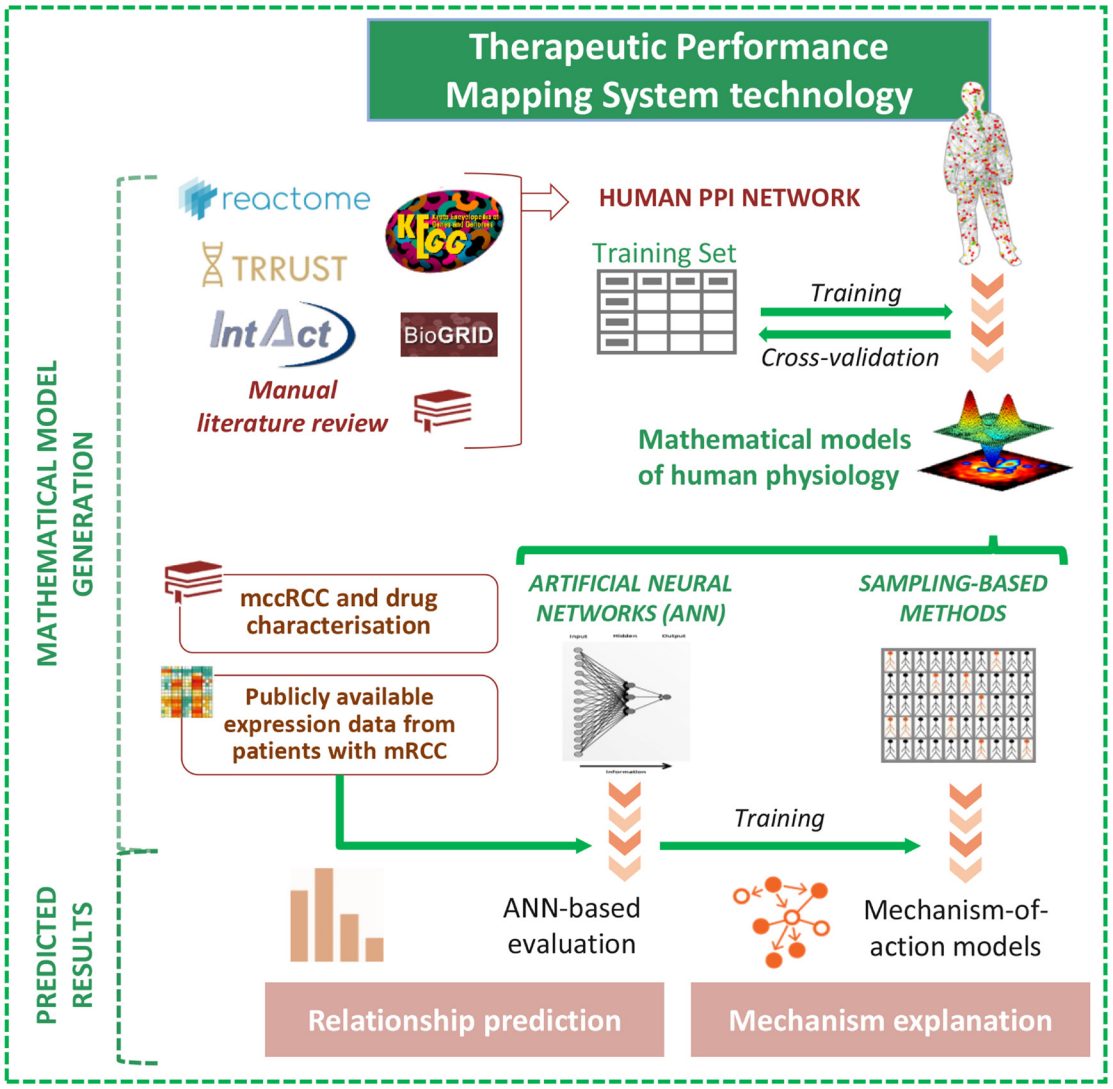
Schematic TPMS approach to analyse the efficacy and the mechanisms of action (MoA) of cabozantinib and/or a PD1 inhibitor in mRCC. TPMS is based on systems biology–based models and encompasses four steps: (i) the learning process of the protein-protein interaction (PPI) human network, based on training and validation with known information stored in the training set; this learning is performed with machine learning techniques to construct accurate mathematical models that simulate the behavior of human physiology through two main strategies: ANNs and Sampling-based Methods. (ii) The molecular characterisation of mRCC disease and drugs, through a comprehensive bibliographical revision, from which mRCC disease interactome can be constructed using the PPI human network. (iii) ANN evaluation of drugs efficacy over mRCC disease definition. (iv) The construction of specific MoA models for mRCC disease and drugs. Abbreviations: ANN: artificial neural network; mccRCC: metastatic clear cell renal cell carcinoma; mRCC: metastatic renal cell carcinoma; PPI: protein–protein interaction.

We constructed a mechanistic systems biology–based model of the effect of cabozantinib combined with PD1 inhibitors in the context of mRCC using the TPMS technology. This integrates available information from human databases to simulate human physiology (Figure 1).

A comprehensive review of the literature allowed to identify five hallmark processes (so-called “motives”) implicated in mRCC pathophysiology: (1) tumour invasion and metastasis, (2) angiogenesis, (3) immune evasion, (4) cell growth and proliferation, and (5) apoptosis evasion. The molecular and functional characterisation of these processes yielded 85, 33, 50, 84, and 31 proteins (so-called effector proteins) associated with each respective motive, with the involvement of 229 non–duplicated effector proteins (Supplementary Table 1). We also characterised the profile of cabozantinib and anti-PD1 protein targets (Supplementary Table 2) to be used for subsequent analyses.

We used two complementary modelling approaches to develop machine learning –based models of patients with mRCC: Artificial Neural Network (ANN), to estimate the probability of a relationship between drugs’ effects and each motive; and Sampling Methods, to describe potential biological mechanisms occurring in mRCC (see Methods). ANN models presented an accuracy of 81.23% with respect to the training set for those drugs with all targets in the HPN, whereas Sampling-based Methods models presented a 94% accuracy.

### Cabozantinib in combination with a PD1 inhibitor provides a wide coverage of mRCC pathophysiological processes that could be behind the clinically observed synergism

The generated ANN model allowed the identification of mathematical and biological relationships between the target proteins of cabozantinib and/or anti-PD1 (Supplementary Table 2) and the effector proteins involved in mRCC pathophysiology (Supplementary Table 1). ANN score values were categorised according to their probability of being true positives, considering *p*-value <0.05 (ANN score ≥78) as high probability, and further categorising lower probabilities (Table 1). *p*-values ≥0.2 (ANN score <47) were considered as negative results and indicators that the ANN does not detect a potential therapeutic effect of this combination upon disease (see Materials and Methods).

**Table 1:** Relationship criteria: ANN scores categorisation and their associated *p*-values

ANN score	Likeliness of the predicted relationship	Associated *p*-value
≥78	HIGH (+++)	<0.05
71–78	MEDIUM-HIGH (++)	0.10–0.05
47–71	MEDIUM (+)	0.20–0.10
<47	LOW (–)	≥0.20

To assess the specific mechanisms underlying the potential synergy of both therapies we screened the ANN scores linking treatments, individually or combined to each pathophysiological motive in mRCC (Table 2).

**Table 2:** Effects of cabozantinib and/or PD1 inhibitor in mRCC by means of artificial neural networks

mRCC pathophysiological processes	Cabozantinib		PD1 inhibitor		Cabozantinib + PD1 inhibitor	
	*p* value^1^	ANN score	*p* value	ANN score	*p* value	ANN score
Apoptosis evasion	**++**	**74**	**+**	**56**	**+++**	**89**
Immune evasion	**+++**	**83**	**+++**	**83**	**+++**	**89**
Angiogenesis	**+++**	**87**	−	5	**+++**	**86**
Metastasis and Invasion	**+++**	**87**	−	20	**+++**	**85**
Cell growth and Proliferation	**+++**	**81**	−	26	**+++**	**81**

Results are expressed as ANN score and categorized according to *p*-values. Shaded cells indicate an additive effect between cabozantinib and the PD1 inhibitor. Significant values (≥47) are shown in bold. ^1^Probability of existence of true relationships between the drugs’ targets and the pathophysiological motives evaluated, according to ANN–based predictive models. Abbreviations: ANN, artificial neural networks. *p*-value correspondence: ANN score ≥78, *p*-val <0.05 (+++, high probability); ANN score ≥71, *p*-val <0.1 (++, medium–high probability); ANN score ≥47, *p*-val <0.2 (+, medium probability), ANN score <47, *p*-val ≥0.2 (–, low probability; see Table 1).

The individual drug assessment suggested that cabozantinib modulates all mRCC pathophysiological processes (Table 2), being most prominently associated to angiogenesis and metastasis (ANN scores 87), but also to immunosurveillance, tumour proliferation, and, with a lower probability, apoptotic mechanisms. Anti-PD1 therapies displayed a more limited mechanistic relationship with mRCC, mainly focused on reactivation of tumour immunosurveillance mechanisms, although a significant relationship was found with apoptosis evasion (ANN score 56). The ANN analysis did not to detect additional mechanistic relationships between PD1 therapies and the remaining processes. On the other side, the addition of anti-PD1 agents to cabozantinib seems to mainly strengthen the mechanistic relationship of the latter on immunosurveillance and apoptotic mechanisms (shaded cells), which is reflected by the higher ANN scores of the combined therapy than those of individual treatments.

A subsequent ANN analysis evaluating the effect of each cabozantinib target (Supplementary Table 2) on mRCC motives was run to identify the cabozantinib targets with a stronger relationship with each motive, and/or presenting an additive effect when combined with a PD1 inhibitor (Table 3).

**Table 3:** Effects of cabozantinib targets, individually (*Cabo*.) and in combination with a PD1 inhibitor (*Comb*.), in relation to mRCC motives by means of artificial neural networks

Cabozantinib targets	Cell growth and proliferation		Apoptosis evasion		Angiogenesis		Immune evasion		Metastasis and invasion	
	Cabo.	Comb.	Cabo.	Comb.	Cabo.	Comb.	Cabo.	Comb.	Cabo.	Comb.
AXL	−(33)	−(33)	−(7)	−(40)	−(30)	−(19)	**+(54)**	**+++(81)**	**+(48)**	**+(63)**
FLT1 (VEGFR1)	−(31)	−(34)	−(9)	−(39)	**+(56)**	**+(59)**	**+(53)**	**+++(79)**	−(29)	−(29)
FLT3	−(33)	−(33)	−(17)	−(38)	−(20)	−(22)	−(19)	++(74)	−(28)	−(29)
FLT4 (VEGFR3)	−(31)	−(34)	−(10)	−(39)	−(32)	−(20)	−(17)	++(73)	−(28)	−(27)
KDR (VEGFR2)	−(43)	−(42)	−(12)	−(40)	**+(58)**	**+(63)**	−(31)	++(72)	−(34)	−(34)
KIT	−(40)	−(43)	−(19)	−(37)	**+(51)**	**+(61)**	−(20)	++(74)	−(31)	−(29)
MERTK	−(24)	−(28)	−(6)	−(38)	−(24)	−(21)	**+(52)**	**+++(81)**	−(28)	−(31)
MET	−(40)	−(38)	−(11)	−(45)	−(23)	−(17)	−(14)	+++(82)	++(77)	++(71)
NTRK2	+(53)	−(44)	−(7)	−(38)	−(22)	−(22)	−(16)	++(74)	**+(49)**	**+(63)**
RET	−(31)	−(31)	**+(49)**	**+(61)**	−(22)	−(22)	−(17)	++(74)	**+(47)**	**+(61)**
ROS1	−(20)	−(31)	−(7)	−(40)	−(22)	−(20)	−(15)	++(73)	−(22)	−(28)
TEK	−(32)	−(31)	−(9)	−(38)	**+(48)**	**+(65)**	−(15)	++(73)	−(18)	−(24)
TYRO3	−(23)	−(31)	−(6)	−(40)	−(21)	−(21)	**+(49)**	**++(77)**	−(32)	−(32)

The data reflect the probability of the existence of true relationships between the drug target proteins and the effector proteins related to the pathophysiological motives, according to ANN–based predictive models. *p*-value correspondence: ANN score ≥78, *p*-val <0.05 (+++, high probability); ANN score ≥71, *p*-val <0.1 (++, medium-high probability); ANN score ≥47, *p*-val <0.2 (+, medium probability), ANN score < 47, *p*-val ≥0.2 (−, low probability). Abbreviations: ANN score; Cabo., cabozantinib; Comb., combination cabozantinib + PD1 inhibitor. Results are expressed as ANN score categorisation, and the ANN score is shown in brackets. Significant values (≥47) are shown in bold. Shaded cells indicate additive effects of cabozantinib and the PD1 inhibitor.

This analysis revealed a poorer mechanistic relationship between each cabozantinib target and the motives defining mRCC disease (Table 3), than when considering the complete drug target profile (Table 2). This suggested a cooperative effect instead of individual contribution of several targets to the overall effect in mRCC. However, these results allowed to identify additive effects between the targets of cabozantinib and PD1 inhibition for angiogenesis and metastasis (Table 3), besides immunosurveillance and apoptotic mechanisms (shaded cells). This comprehensive analysis was used to identify those cabozantinib targets with a potentially stronger relationship (ANN score >47) with each mRCC pathophysiological motive showing an additive effect with a PD1 inhibitor (Table 3, shown in bold, and Table 4).

**Table 4:** Cabozantinib targets with a potential relationship to each pathophysiological pathway/motive showing additive effects in combination with anti-PD1

	Cabozantinib target/s with positive predicted relationship^1^ and subject to additive effects^2^ of a combined therapy with anti-PD1
Cell growth and Proliferation	-
Apoptosis evasion	RET
Angiogenesis	FLT1 (VGFR1), KDR (VGFR2), KIT, TIE2 (TEK)
Immune evasion	AXL, FLT1 (VGFR1), MERTK, TYRO3
Metastasis and Invasion	AXL, NTRK2 (TRKB), RET

^1^ANN score >47, ^2^ANN score (combination) >ANN score (individual drug target).

### Cabozantinib combined with a PD1 inhibitor were predicted to collaboratively modulate the complex interplay between multiple pathways, cells, and molecules of the mccRCC tumor microenvironment

#### The combined treatment is predicted to boost immunosurveillance mechanisms, and impair pro–angiogenic microenvironment

To trace the most relevant plausible additive paths between treatments in the cited motives, we applied the Sampling Methods modelling approach. This allows to assess the ability of each treatment to reverse the protein alterations reported in the mRCC molecular characterisation.

**Figure 2:**
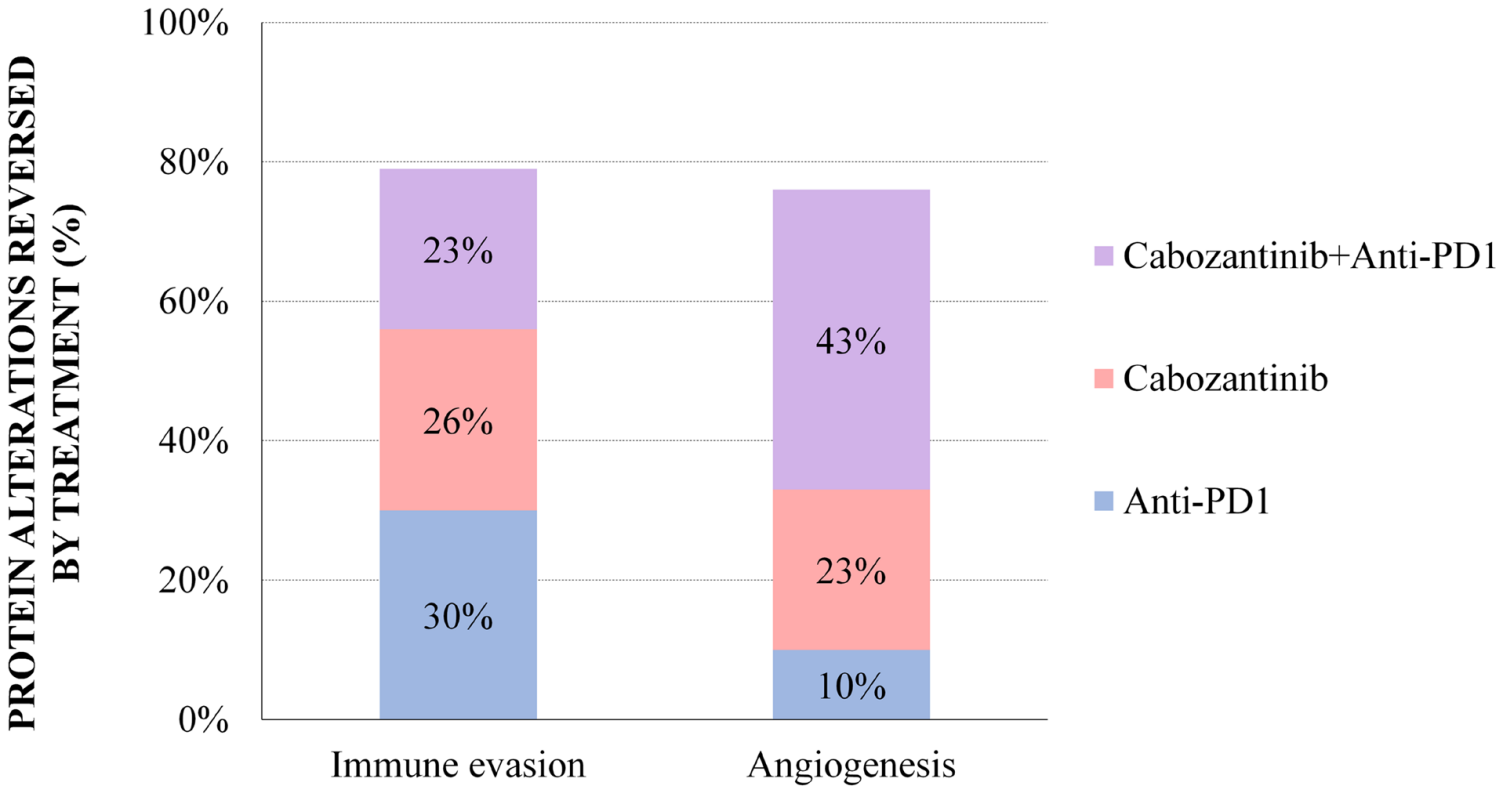
Sampling Methods additive models: percentage of mRCC effector proteins reversed by treatment in the angiogenesis and immune evasion motives, for which additive mechanisms were detected both by ANN and Sampling-based Methods. The percentages reflect the proportion of mRCC–associated protein alterations that are reversed by one or both treatments. The detail on the specific proteins modulated by each treatment is provided in Supplementary Tables 3 and 4.

First, a combinatorial model —anti-PD1+cabozantinib defined as the targets most probably related to each motive, contributing to the additive effect (Table 4)— was constructed for the four motives for which an additive effect had been detected by ANN analysis: immunosurveillance evasion, apoptosis evasion, angiogenesis, and metastasis and invasion. However, through the Sampling Methods strategy, additive mechanisms were only detected and traced for immunosurveillance evasion and angiogenesis (Figure 2). In line with our ANN model (Table 2), the combined pharmacological action of both drugs in mRCC tumours was shown to achieve a wider coverage of the immunosurveillance evasion mechanisms than either cabozantinib or anti-PD1 compounds alone: 23% overlapping effects, and 26% and 30% compound-specific effects for cabozantinib and anti-PD1, respectively (Figure 2, Supplementary Table 3).

This also applies to the tumour pro–angiogenic mechanisms, which are modulated by 43% overlapping drug effects, with a lower PD1 inhibitor specific contribution in this case (10%; Figure 2, Supplementary Table 4). Though the ANN model was unable to detect a biological relationship between angiogenesis and anti-PD1 therapy (Table 2), a boosting effect was uncovered when combining some cabozantinib targets with PD1 inhibition (Table 3). The Sampling Methods mechanistic approach further enabled to detect several proteins involved throughout the whole angiogenesis process, affected either by the anti-PD1 blockade, and by cabozantinib (Figure 2, Supplementary Table 4). All in all, these results illustrate the boosting effects of the combination over the individual drugs to reduce immune evasion and inhibit the pro–angiogenic microenvironment in mRCC disease.

**Figure 3:**
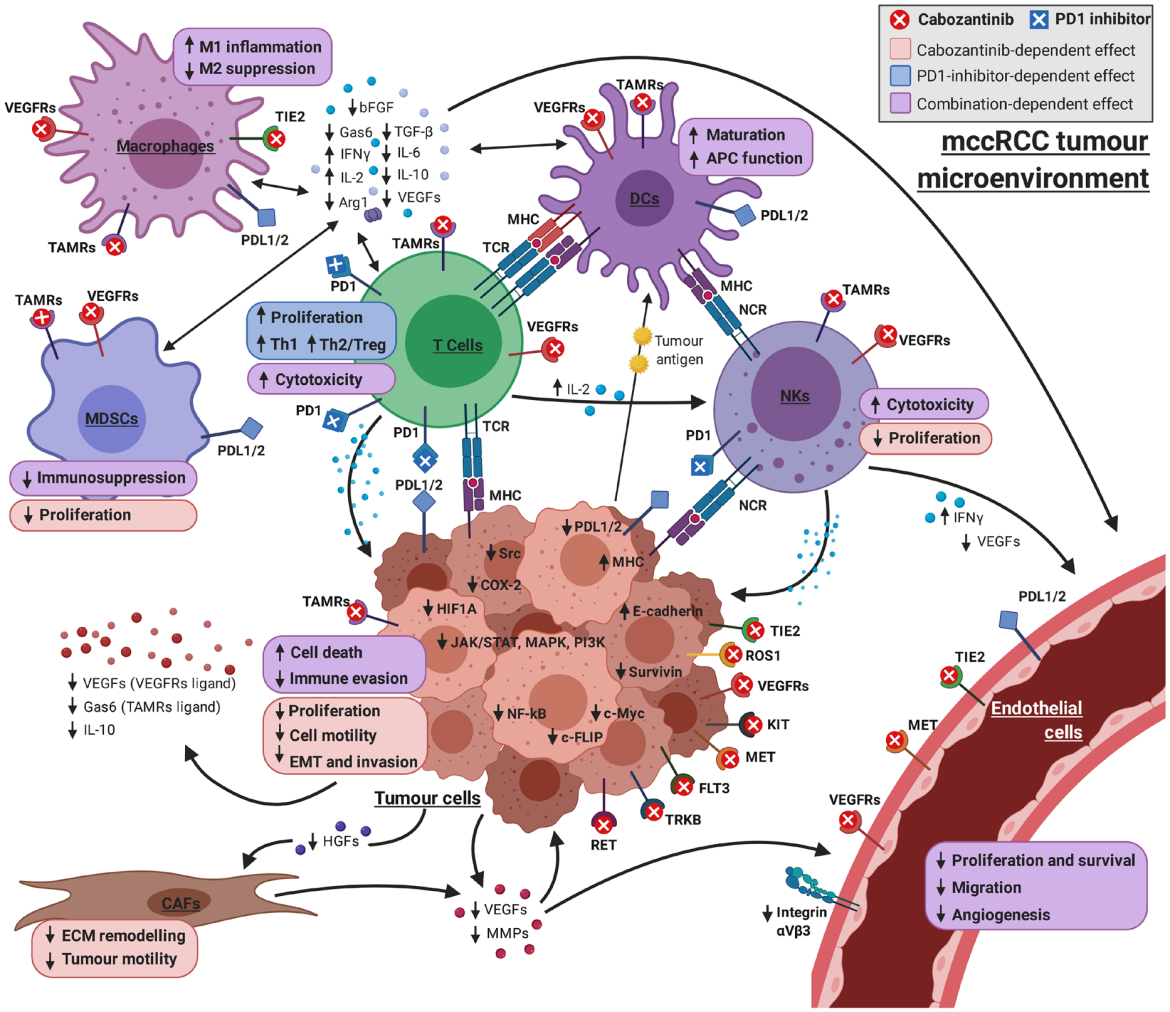
Graphical overview of the mechanism of cabozantinib + PD1 inhibitor on the complex interplay between the cell types involved in mRCC pathogenesis. The combination of drugs has a collaborative effect on immunosurveillance, commonly affecting immune cells, such as macrophages, DCs, NKs, MDSCs and T cells. Cabozantinib has a direct role in apoptosis, targeting tumoural cells, but also enhancing pro–cytotoxic effects of PD1 inhibitors on T-cells. Cabozantinib direct effect on vascular endothelial cells through tyrosine kinase inhibition is combined with the modulation of the microenvironment by both drugs, reducing the availability of the pro–angiogenic factors produced both by tumoural and immune cells (VEGFs, FGF, cytokines, MMPs…). Finally, cabozantinib can prevent proliferation and invasion mechanisms by targeting tumoural cells, vascular endothelial cells and CAFs. Model-derived mechanisms and bibliographical references supporting each link can be found in Supplementary Material (Supplementary Figures 1–5 and Supplementary Tables 5–9). Picture created with https://BioRender.com.

The proteins affected by the drugs and involved in these mechanisms exert their function in several cell types (Table 5 and Figure 3). The mechanistic details and proteins involved are displayed in Supplementary Figures 1, 2 and Supplementary Tables 5, 6, respectively.

**Table 5:** Detail of proteins modulated by cabozantinib and a PD1 inhibitor, and their effect on different cell types

Processes modulated by cabozantinib and a PD1 Inhibitor			
Cell Type	Process	Effects	Effectors
**Tumour cells**	Immune evasion	Reduction of the immune-evasive gene expression programme	↓ HIF1A, GAS6
	Angiogenesis	Inhibition of tissue remodelling and neovascularisation	↑ VEGFA, HIF1A ↓ MMP2, MMP9
**T-cells**	Immune evasion	Immunosurveillance activation, pro–inflammatory phenotype cytotoxic response against the tumour	↑ IL-2, IFNG ↓ SMAD3, EZH2, VEGF
	Angiogenesis	Increase of anti–angiogenic agents, inhibition of tissue remodelling	↑ IFNG ↓ MMP9, VEGF, bFGF
**MDSC**	Immune evasion	Inhibition of the immunosuppressive phenotype	↓ IL-10, ARG1
**DC**	Immune evasion	DC maturation	↓ IL-10, GAS6, IL-6
**Macrophages**	Immune evasion	M1 macrophage polarisation	↓ IL-10, HIF1A, GAS6, EZH2
**NK cells**	Immune evasion	NK cell commitment into a cytotoxic phenotype	↑ IFNG, ↓ EZH2
**Endothelial cells**	Angiogenesis	Reduction of vascularization and endothelial cell migration	↓ VEGF, αVβ3

^1^Abbreviations: ARG1: arginase 1; bFGF: basic fibroblast growth factor; CAF: cancer-associated fibroblast; EZH2: enhancer of zeste homolog 2; DC: dendritic cells; GAS6: growth arrest–specific protein 6; HIF1: hypoxia inducible factor; IFNG: interferon gamma; αVβ3: integrin αvβ3; IL: interleukin; MDSC: myeloid-derived suppressor cells; MMP: metalloproteinase; NK: natural killer; PD1: programmed cell death protein 1; SMAD3: mothers against decapentaplegic homolog 3; VEGF: vascular endothelial growth factor.

In particular, the beneficial collaboration between drugs seems to boost immunosurveillance mechanisms in the tumour microenvironment, modulated mainly through simultaneous PD1 blockade and inhibition by cabozantinib of Tyro3/AXL/MER (TAM) receptors and VEGFR1. This results in an inhibitory effect on immunosuppressor factors, such as IL10, IL6, GAS6, ARG1, EZH2 or HIF1A; and activation of immunostimulatory molecules, including IFNG and IL2, over a plethora of cell types in the tumour microenvironment (Supplementary Figure 1, Supplementary Table 5, Table 5, Figure 3).

The model also predicts a decline of the pro–angiogenic microenvironment, primarily through cabozantinib inhibition of VEGF receptors 1 and 2, angiopoietin receptor TIE2 and cKIT, in partial collaboration with anti-PD1 agents (Supplementary Figure 2, Supplementary Table 6, Table 5, Figure 3). Downstream effects include downregulation of important pro–angiogenic factors, such as VEGFA, HIF1A, MMP2, MMP9, COX2, bFGF or integrin αVβ3, and upregulation of the antiangiogenic molecule IFNG. These effects involve immune cells and vascular endothelial cells in the tumour microenvironment, as well as tumour cells.

#### Cabozantinib is predicted to hamper tumour proliferation, motility and invasion, and apoptosis

The biological rationale behind the collaborative mechanism through which anti-PD1 agents promote tumour cell apoptosis and metastasis, as revealed by our ANN model (Tables 2 and 3), was proven difficult to simulate through our Sampling Methods–based approach (data not shown). In the case of apoptotic mechanisms, this is probably due to the indirect nature of this effect: while cabozantinib has a direct effect on inducing apoptosis in tumour cells, PD1 inhibition indirectly promotes apoptosis through T-cell induced cytotoxicity on the tumour cells, i.e. eliciting the immune response that prevents the tumour immune evasion (Figure 2, Table 5).

**Figure 4:**
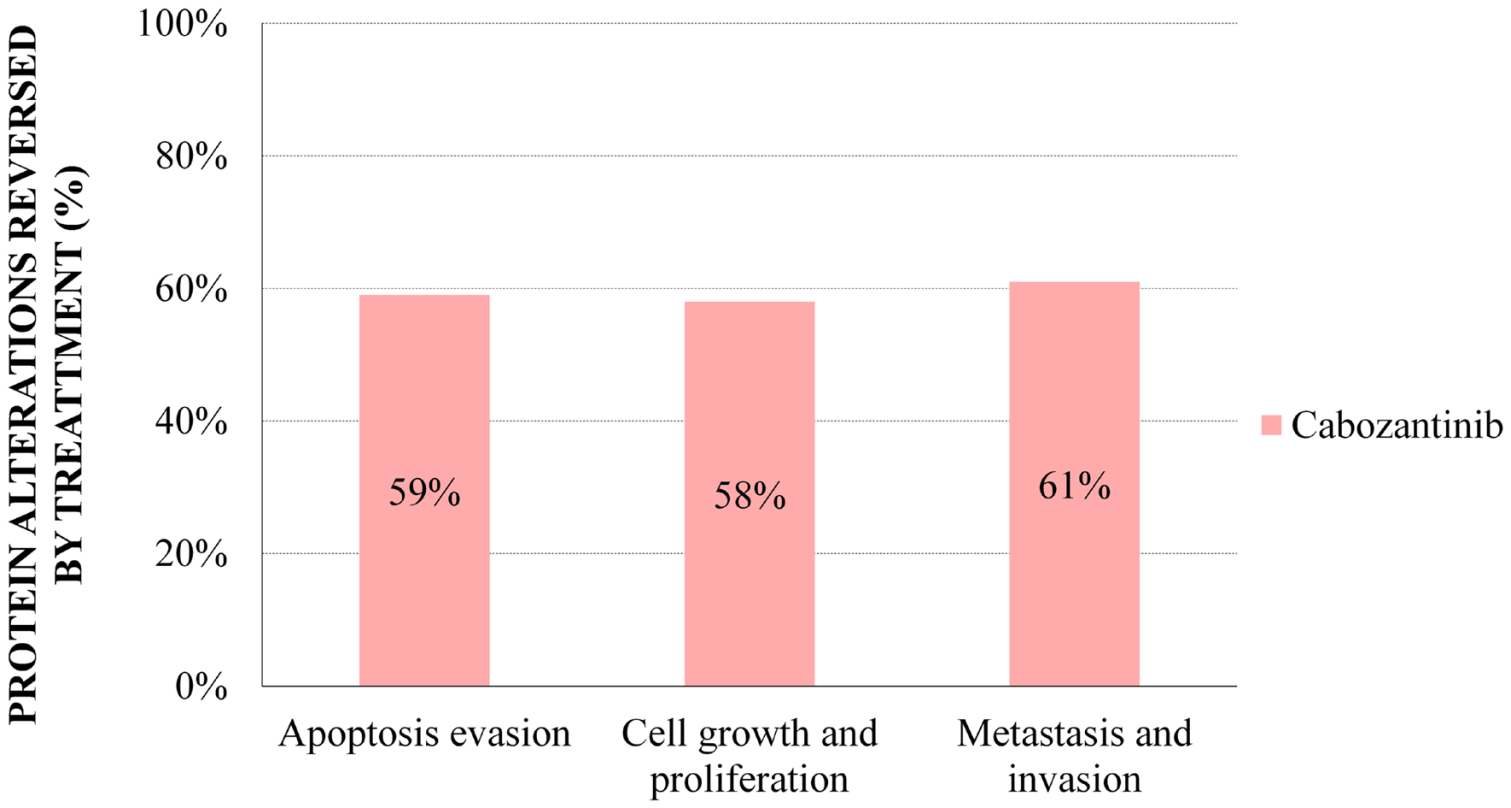
Sampling Methods cabozantinib models: percentage of mRCC effector proteins reversed by treatment with cabozantinib. The percentages reflect the proportion of mRCC–associated protein alterations that are reversed by cabozantinib according to Sampling Methods models.

Focusing on the motives that had failed to show additive effects between the agents according to the ANN analysis, or no additive effects were detected in the Sampling Methods-based models, we used a cabozantinib–centered Sampling Methods model to assess its individual impact on these mRCC motives (Supplementary Figures 3–5, and Supplementary Tables 7–9). Apart from its association with apoptosis evasion processes (Figure 4), and in line with the previous ANN analysis (Table 2), cabozantinib was shown to reverse nearly 60% of protein effectors involved in proliferation and cell growth, and tissue invasion and metastasis in mRCC (Figure 4). These results show that cabozantinib impairs proliferative signals, invasive and migration properties, and apoptosis evasion (Table 6, Figure 3).

**Table 6:** Detail of proteins modulated by cabozantinib, and their effect on different cell types

Processes modulated by cabozantinib			
Cell type	Process	Effects	Effectors
**Tumour cells**	Apoptosis evasion	Programmed cell death of tumour cells	↓NF-κB, c-FLIP, VEGF
	Cell growth and proliferation	Reduction of altered proliferation signals	↓ MAPKs, JAK-STAT, PI3K, SRC
	Tissue invasion and metastasis	Reduction of invasive and migratory properties (cell adhesion)	↓ MMP2, HIF1A, CXCR4 ↑ E-cadherin
**T-cells**	Apoptosis evasion	Cytotoxic response against the tumour (*Indirect effect by boosting immunosurveillance*)	*(VEGFR1, TYRO3, AXL, MER)*1
**Endotelial cells**	Tissue invasion and metastasis	Reduction of vascularisation and endothelial cell migration	↓ VEGF, HIF1A
**CAF**	Tissue invasion and metastasis	Reduction of extracellular matrix remodelling, tumour cell invasiveness and motility	↓ SDF1, MMP2, HGF

^1^Parentheses reflect an indirect effect of cabozantinib that could boost PD1 effects. Abbreviations: AXL: Tyrosine-Protein Kinase Receptor UFO; CAF: cancer-associated fibroblast; c-FLIP: cellular FLICE (FADD-like IL-1β-converting enzyme)-inhibitory protein; CXCR4: CXC chemokine receptor 4; DC: dendritic cells; HGF: hepatocyte growth factor; JAK: Janus kinase; MAPKs: mitogen-activated protein kinase; MDSC: myeloid-derived suppressor cells; MER: Proto-oncogene tyrosine-protein kinase MER; MMP: metalloproteinase; NK-kB: nuclear factor kappa B; PD1: programmed cell death protein 1; PI3K: phosphoinositide 3-kinase; SDF1: stromal cell-derived factor 1; SRC: Proto-oncogene tyrosine-protein kinase Src; STAT: Signal Transducer and Activator of Transcription; TYRO3: Tyrosine-protein kinase receptor TYRO3; VEGF: vascular endothelial growth factor.

Pro–apoptotic signalling induction is exerted in tumour cells through inhibition of ligand–RET receptor interaction by cabozantinib (Table 3), among other cabozantinib targets (Supplementary Figure 3, Supplementary Table 7, Table 6, Figure 3). These predicted effects rely in the inactivation of key transcription factors (NF-κB, c-FLIP, VEGFA, survivin or BIRC5) involved in resistance to death and inhibition of survival signals in cancer cells.

Cabozantinib monotherapy was predicted to decrease migration and invasion properties of tumour cells and restore cell adhesion. This entailed inhibition of TYRO3 and multiple master regulators of the metastatic process, such as the proto-oncogene SRC complex (FYN), PI3K and STAT3. Ultimately, it prevented nuclear translocation of transcription factors (HIF1A), promoted E-cadherin activity, and reduced expression of proteins characteristic of an invasive phenotype (MMP2, CXRC4, and heparinase/HPSE; Supplementary Figure 4, Supplementary Table 8, Table 6, Figure 3).

Cabozantinib was also predicted to impair proliferative signals in mRCC tumours through effectors involving the MAPK–kinase family, JAK-STAT, PI3K and ERK pathways, and proto–oncogenes such as c-SRC. Their inhibition is key to prevent the transcription of multiple nuclear factors that otherwise would restore the cell cycle (Supplementary Figure 5, Supplementary Table 9, Table 6, Figure 3).

According to our model, both treatment strategies, cabozantinib and anti-PD1, modulate proteins with a role in tumour cells and the tumour microenvironment including T-cells, myeloid-derived suppressor cells, dendritic cells, macrophages, natural killer cells and vascular endothelial cells. On the other hand, cabozantinib exerts its effect inhibiting proteins involved in regulation of extracellular matrix (ECM) remodelling and tumour invasiveness and motility (SDF1, MMP2 and HGF) by cancer-associated fibroblasts (CAFs; Table 3, Tables 5–6, Figure 3, Supplementary Figures 1–5, Supplementary Tables 5–9).

## DISCUSSION

This is the first study to describe the potential mechanisms underlying the synergic effects of a therapy combining cabozantinib plus a PD1 inhibitor in mRCC through novel systems biology– and machine learning –based techniques. The results suggest that the combination therapy provides a wider coverage of mRCC pathological mechanisms and a greater therapeutic effect than each separate treatment. The synergy may occur mainly in the immune evasion domain, providing beneficial effects to overcome mechanisms driving immunosurveillance evasion in mRCC tumours. We also identified synergic contributions of PD1 blockade to the known anti–angiogenic and tumour pro–apoptotic effects of cabozantinib, lessening the angiogenic microenvironment through immune modulation and modulating T-cell cytotoxicity. The combination creates a therapeutical effect in the tumour and the microenvironment that encompasses multiple cellular types, allowing the treatment to modulate the full spectrum of mRCC physiopathology. Additionally, cabozantinib participates in hampering anti–apoptotic and proliferative signalling and invasion properties and contributing to restore cellular adhesion in tumour cells.

### Effects in tumour immunosurveillance

Our model predicts boosted immunosurveillance for the combined therapy. Thus, inhibition of VEGF-VEGFR and Gas6-AXL/TYRO3/MER (TAM) axes by cabozantinib modulates humoral and cellular components of the innate and adaptative immune responses inducing immunostimulatory phenotypes. Decreased HIF1A activity hampers the immune-evasive programme and enhances tumour recognition by T-cells [11, 25]. TAM and PD1 blockade can simultaneously modulate the activity of several immune cells: switching from pro–tumour M2 to pro–inflammatory M1 macrophage polarization [26–29], and increasing immune response by dendritic cells (DC) [26, 30] and NK cells [31, 32].

In addition, PD1 blockade disables downstream effectors involved in TME immunosuppression including the IL10 cytokine [33], which blocks antigen presentation capabilities of antigen-presenting cells (APCs) [34]; or the arginase 1 (ARG1), whose upregulation in MDSC provokes non–specific T-cell inhibition [35].

Likewise, anti-PD1 would induce expression of immunostimulatory molecules like IFNG and IL2 in NK cells [33] and T-cells [36], critical cytokines for effective immune responses in RCC [37].

PD1 blockade in T-cells has more immunosurveillance consequences, as PD1-PDL1 engagement restrains their proliferation via SMAD3 [38]. Anti-PD1 could return these T-cells into a proliferative phenotype, downregulate apoptotic pathways and promote their survival and TCR-mediated activation [10, 39], increasing their tumour destruction capability [8, 40].

### Effects in angiogenesis

Our model predicted a potential combinatorial effect between PD1 inhibitors and VEGFR1, VEGFR2, KIT and TEK/TIE2 cabozantinib targets in impairing angiogenesis.

Anti-PD1 would reinforce cabozantinib antiangiogenic effects by modulating pro–angiogenic and antiangiogenic factors. In this context, PD1 blockade in T-cells enables ERK1/2 to activate STAT1 and SMAD3 [40, 41], inhibiting MMP9 [42] and bFGF [43] —an agonist of VEGFR1/VEGFR2/TIE2 angiogenic receptors. MMP9 controls VEGF release from tumour and neighbouring cells in the metastatic niche [44], so its decline would mitigate the angiogenic switch [45]. On the other hand, PD1 blockade upregulates interferon IFNG [41], a potent antiangiogenic molecule [46].

In the tumour cells, cabozantinib interrupts VEGFR1/VEGFR2/c-KIT/TIE2 pathways [47], downregulating angiogenic factors (HIF1α, VEGFA) [48] and vascular remodelling factors (MMP2, MMP9) [49].

In endothelial cells, impaired VEGFR1/VEGFR2 activation by cabozantinib downregulates angiogenic factors. VEGFA secretion maintains endothelial cell viability in the tumour surroundings [50]. c-Myc inactivation —due to RTKs intracellular signalling or to T-cell secreted IFNG signalling upon PD1 blockade [51]— might reduce VEGFA and other angiogenic factors, and diminish endothelial cell proliferation [46]. Also, VEGFR2 inactivation downregulates the reciprocal activation between this receptor and integrin αVβ3, which regulates key vascularisation processes [52].

The simultaneous modulation of cell types and molecules (bFGF, IFNG, MMP9) might underlie the potential synergy detected between treatments targeting angiogenesis.

### Effects in apoptosis evasion

Our model predicted potential combinatorial effects between anti-PD1 and cabozantinib in apoptosis evasion, particularly through RET. However, the Sampling-based Methods approach did not detect those combinatorial mechanisms but detected pro–apoptotic mechanisms of cabozantinib targets. These cabozantinib effects can potentiate the anti-PD1–induced cytotoxic T-cells effects, and this collaboration indirectly increases T-cell cytotoxicity [8, 10, 39, 40]. In a strict sense, this anti-PD1 therapy contribution to tumour apoptosis would be more related to immunosurveillance.

In addition, cabozantinib was predicted to exert a direct tumour proapoptotic induction through inhibition of several receptors, including RET. This proto-oncogene promotes cell survival [53] and its inhibition induces apoptosis and prevents growth of some cancer types [54]. RET downstream pathways converge in STAT3 and NF-κB, and the regulation of genes associated with resistance to apoptosis. Among them, c-FLIP participates in tumour progression correlates with poor prognosis [55]; and VEGFA contributes to cell growth and malignant transformation [56, 57].

### Effects in invasive and migratory properties

Cabozantinib–mediated inhibition of RTKs exerts important modulatory effects on tumour cells and the TME. While some additive effects were detected by ANN models between AXL, TRKB and RET targets of cabozantinib and PD1 inhibition, no Sampling Methods models could identify the specific additive mechanisms.

In our model, cabozantinib inhibitory effect on metastasis was shown by preventing the release of proteins involved in the invasive phenotype and attenuation of cellular adhesion. ERK1/2 signalling blockade hampers matrix remodelling, cell motility and metastasis [26, 58] and mitigates the invasive potential of mRCC cells, regulating Snail/SNAI1, Slug/SNAI2 and MMPs, and restoring E-cadherin function [59, 60]. NF-κB inactivation abrogates its suppressive effect on E-cadherin [61] and the expression of SNAI1/2 [62], extracellular chemokines (SDF1/CXCR4) [63] and matrix degradation molecules such as heparanase/HPSE and MMP2 [64]. Finally, increased cytoplasmic ß-catenin in RCC results in reduced cadherin-based cell adhesivity and consequent increase in epithelial mesenchymal transition (EMT) with progressive cancer metastases [65, 66].

Cabozantinib would also diminish the involvement of tumour adjacent cell types by preventing remodelling of the TME into an invasion prone state. Thus, restraining the secretion of MMP2 and CAFs activation inducers by tumour cells [67], cabozantinib would avoid the release by CAFs of pro–motility (SDF1) or ECM remodelling-stimulating (HGF) factors [68]. Also, on vascular endothelial cells, cabozantinib would prevent HIF1A modulation, inhibiting tumour invasion [69].

### Effects in cellular proliferation

While no additive effects were detected for the combination regarding cellular proliferation, this motive was predicted to be reduced by cabozantinib through a decline of pro–survival signals in mRCC tumours. Pathways involved in proliferation attenuated by cabozantinib include the MAPK kinase family, PI3K-AKT, JAK/STAT and ERK cascades [58, 70], with the SRC complex coordinating growth and survival signals [71].

### Results contextualization regarding nonclinical and clinical data

Nonclinical [72, 73] and clinical studies [74] point to a synergistic antitumor activity of cabozantinib combined with a PD1 inhibitor in patients with mRCC [11, 14], but just a few have delved into the biological basis of the effect. Primary and metastatic prostate mouse tumour models revealed synergistic efficacy when combining cabozantinib with a dual checkpoint inhibitor therapy (CPI, anti-PD1/anti-CTLA-4) that inactivated MDSCs suppressive activity on cytotoxic T-cell proliferation. [72]. The combination elicited reduced tumour growth, pronounced apoptosis and antitumour immunity that suggested an impact of cabozantinib on the TME hampering immunosuppressive MDSCs activity. In another study, simultaneous treatment with anti-PD1 and anti-VEGFR2 in a murine colon cancer model synergistically inhibited tumour growth [73].While PD1 blockade had no impact on angiogenesis, several proinflammatory cytokines were overexpressed, suggesting an effect in T-cell infiltration into tumours and enhanced immune activation. Cabozantinib had shown to mediate both tumour sensitivity to immune–mediated killing and altered immune landscape. In the study, cabozantinib altered the phenotype of murine tumour cells, sensitising them to immune-mediated killing. Cabozantinib, also synergized with an immunotherapy cancer vaccine to modulate immune subpopulations in the TME, improving T-cell proliferation and infiltration, reducing tumour vascularity and growth-rate, and reducing MSDC and tumour–associated macrophages infiltration [12]. Clinical studies have also demonstrated the immunomodulatory activity of cabozantinib: myeloid cells phenotype was switched from immunosuppressive to antitumor in patients with renal cell carcinoma (RCC), accompanied by an increase in cytotoxic NK- and T-cells [75]. In patients with metastatic urothelial carcinoma cabozantinib treatment reduced peripheral Treg cells [76]. Cabozantinib increased cytotoxic T-cells while reducing peripheral MDSCs in a phase 2 trial of triple-negative breast cancer patients [77]. A phase 1b study of castration-resistant prostate cancer (CRPC) showed that cabozantinib/atezolizumab combination was associated with an increment in the number of activated cytotoxic T-cells accompanied with a decrease in immunosuppressive cells in peripheral blood [78]. Our model is consistent with these results, as cabozantinib was predicted to hamper the EMT and the combination was predicted to modulate the TME through a pro–angiogenic microenvironment decline, immunosurveillance activation, and proapoptotic signalling induction in tumour cells, antiapoptotic in T-cells.

### Challenges, limitations and advantages

Addressing mechanistically tumour complexity and response to therapies is challenging. In addition to the genomic landscape [17] and the intra–tumour molecular heterogeneity characteristic of ccRCC [79], the complex interplay among TME components —tumour, immune and endothelial cells, structural and extracellular matrix molecules, and stromal cells, among others [2, 7]— orchestrate tumour progression and treatment response. Combinatorial therapy exacerbates this complexity, considering that each cellular or molecular component participating in the mechanism of one drug may impact on other pathways and cell types and contribute to the effect of the other drug, and vice-versa [80]. Likewise, detecting indirect effects involving mediators or come to conclusions is challenging. The present approach successfully addresses this complexity, although not without biological and technical limitations [20]. In our study, we were able to detect and describe potential synergistic effects in immunosurveillance and angiogenesis. However, although the indirect and double–edged nature of the antiapoptotic effect of the combination in T-cells and the proapoptotic effect in tumour cells could be unveiled by the ANN methodology, we could not simulate these mechanisms through Sampling Methods. Similarly, we were unable to identify the additive mechanisms detected for invasion and metastasis through ANN models. This happens when several intermediate molecules are involved generating a mathematically dispersed signal through the protein network that can be overlooked.

The accuracy to simulate RCC pathophysiology is limited by the data about diseases and drug availability in public repositories: unknown targets or processes not yet described cannot be considered. Furthermore, there could be other variables that might affect the results, such as tumour intra– and inter–individual variability, tumour immune phenotype, drug resistance, lack of response, or severe toxicities. While new prospective data might reduce the bias due to the information gap, improve the models, and allow to validate conclusions, the TPMS technology relies on comprehensive biological information on a wide range of drugs and diseases not restrained to RCC or oncologic indications [18–20]. Cross–validation accuracy surpasses 80% in ANN models and 94% in Sampling Methods models, allowing to infer assumptions from one field to another. This enables to create accurate models where molecular information is scarce or the number of study patients is low.

Regarding technical limitations, the ANN methodology detects drug–target relationships based on the algorithm feed of known relationships. In fact, our ANN model was trained with drugs, not individual targets. Although the training process considers the number of targets of each drug for the ANN score generation, the evaluation of individual targets could have been underestimated. In this sense, we used less restrictive criteria to suggest potential relationships when evaluating individual targets. On the other hand, a gap between both definitions concerning angiogenesis and anti-PD1 precluded mathematical model from detecting a true relationship. We overcame this issue appealing to the Sampling Methods, and evaluation of the signal propagation through the protein network provided a measurable impact of the anti-PD1 on angiogenesis target proteins. Thus, we took advantage of the use of two complementary techniques. The technical problem with the Sampling Methods is the abovementioned limitation to detect indirect effects when several effectors are involved and the signal disperses, losing sensitivity.

### Future perspectives

Though our ANN results support a differential role for cabozantinib due to its multi-target profile regarding each individual target evaluation, we cannot affirm whether our results are true for other drug combinations involving antiangiogenics and CPI. Development of similar models to allow mechanistic comparisons of the effect of other TKI in immunomodulatory combinations on mRCC could be a future area of research. Also, the mRCC and therapeutic mechanism models created might uncover potential biomarkers or new targets, so each one could be subject to further investigations.

Given the variety of combination therapies already approved and the ongoing trials, there are several issues that hinder interpretation [6]: most trials benchmarked against sunitinib, intra– and inter–tumour heterogeneity, toxicities, short follow-up studies, among others. Moreover, some trials are investigating triple combinations such as ipilimumab/nivolumab/cabozantinib or involving HIF inhibitors with CPI and TKIs (NCT04736706). New targeted therapies and other immunotherapies are also being tested [3]. In this context, AI technology seems an ideal strategy to explore at a reduced cost the best combinatorial mechanisms in the pre–clinical setting by integrating all variables possible: disease, drug, patient characteristics, tumour type, sequence administration or potential resistance patterns. The greater understanding of how tumour microenvironment modulates mRCC disease and how it can be therapeutically fine-tuned with drug combinations will provide clues to better stratify patients, mitigate toxicities or identify biomarkers and potential therapeutic targets. The hypotheses raised from the mechanistic analyses should be subjected to experimental validation before their implementation into the clinical practice.

## MATERIALS AND METHODS

### Introduction to TPMS technology: mRCC systems biology-based model

Therapeutic Performance Mapping System technology [18] (TPMS; Anaxomics Biotech, Barcelona, Spain) provides an insight into the physiological effects of pharmacological compounds or biological processes at the molecular level, bridging molecular and clinical worlds. Using systems biology and machine learning and pattern recognition techniques, TPMS simulates *in silico* normal and pathological human physiology through mathematical models, integrating comprehensive updated biological, pharmacological and medical knowledge. Using this methodology, described and applied for other diseases [19, 20, 23, 24, 81], we have modelled the pathophysiology of metastatic renal cell carcinoma (mRCC) and screened the targets of both cabozantinib and PD1 inhibitors, alone or in combination (Figure 1).

### Bibliographically-based molecular characterisation

#### Metastatic renal carcinoma characterisation

Pathogenesis and pathophysiology of mRCC or aRCC were comprehensively characterized through an extensive manual curation of the scientific literature, with a detail in the molecular and cellular pathways involved in the biological processes of interest. Only English-language articles were included. Review articles from November 11th 2009 to November 11th 2019 were searched in the PubMed database as follows: *“renal cell carcinoma” [Title] OR “RCC” [Title] OR “metastatic renal cell carcinoma” [Title] OR “mRCC” [Title] OR “advanced renal cell carcinoma” [Title] OR “aRCC” [Title]) AND (pathogenesis [Title/abstract] OR pathophysiology [Title/abstract] OR molecular [Title/abstract].* An analysis of titles and abstracts, followed by a review of the full texts comprising molecular information of interest, allowed the identification of the main pathophysiological processes (referred to as “motives”; Supplementary Table 1) involved in the disease. The search was expanded using relevant references listed in the reviewed articles.

Each motive was further characterised at the protein level (Supplementary Table 1). The publications retrieved were used to screen protein/gene candidates as condition effectors by the association of their functional activity —or lack thereof— with disease development. If scientific evidence for a potential candidate was not sufficiently consistent, an additional PubMed search was performed in the Uni-ProtKB database including the whole array of protein names. Novel candidates identified at this step were added to the list of effectors, following the same criteria and protocol. To ensure a complete molecular characterisation of the disease pathophysiology, general information concerning RCC was included when a specific search on mRCC did not retrieve any results. By February 2020, the disease characterisation had been completed. Previous models created through the same process have yielded experimentally validated conclusions [24].

#### Characterisation of drug targets

For drug molecular definition in mRCC, drug targets were identified through a revision of official documents —European Public Assessment Report (EPAR, European Medicines Agency); Multidisciplinary review and Chemistry review, Food and Drug Administration; Product Monograph—, specialised databases, including DrugBank [82, 83] and Stitch [84] (entries for PD1 inhibitors were found only in DrugBank), and scientific literature in PubMed (Supplementary Table 2). Titles and abstracts were screened, and subsequently reviewed if molecular information was found, to identify proteins/genes as potential drug target candidates, and/or determine pharmacokinetic information.

In the case of cabozantinib, we identified in PubMed updated reviews of known targets of the drugs of interest published in the 5 years prior to December 2nd, 2019, using the following search strings: *“Cabozantinib” [Title] OR “BMS 907351” [Title] OR “BMS907351” [Title] OR “XL 184” [Title] OR “XL-184” [Title] OR “XL184” [Title] OR “Cometriq” [Title] OR “Cabometyx” [Title]) AND (“target” OR “activity assay” OR “binding assay” OR “inhibitor”*. In the case of PD1 inhibitors, although being monoclonal antibodies, specific searches were performed to confirm absence of off-targets: Nivolumab: *(“Nivolumab” [Title] OR “BMS-936558” [Title] OR “GTPL7335” [Title] OR “MDX-1106” [Title] OR “ONO-4538” [Title] OR “Opdivo” [Title]) AND (“PD1” OR “PDCD1” OR “PD-1” OR “Programmed cell death protein 1”) AND (“target” OR “activity assay” OR “binding assay” OR “antibody”); Pembrolizumab: (“Pembrolizumab” [Title] OR “Lambrolizumab” [Title] OR “Merck 3475” [Title] OR “Merck-3475” [Title] OR “Merck3475” [Title] OR “Sch 900475” [Title] OR “SCH-900475” [Title] OR “Keytruda” [Title]) AND (“PD1” OR “PDCD1” OR “PD-1” OR “Programmed cell death protein 1”) AND (“target” OR “activity assay” OR “binding assay” OR “antibody”)*. These searches confirmed target specificity for PD1 (Q15116) (Supplementary Table 2).

#### Expression data analysis

Gene expression data regarding the condition of interest were identified in Gene Expression Omnibus (GEO) public repository [85]. In December 2019, the following queries were performed: *(“Clear cell renal cell carcinoma” [ALL FIELDS] OR “ccRCC” [ALL FIELDS]) AND (“metastasis” [ALL FIELDS])*. Data were filtered by organism (*Homo Sapiens*), by entry type (series) and by experiment type (expression profiling by array); only experiments evaluating different patients and tissue samples from normal kidney and metastatic sites were considered.

Data contributed by Nam [86] (GSE105261) complied with those filters, and metastatic samples were compared to controls using GEO2R [85]; significance criteria was set at adjusted *p*-value <0.01 and |logFC| >2. UniProtKB codes were retrieved from UniProtKB [87] from Gene ID, using the Retrieve tool. Transcripts encoding the same protein were checked to discard those proteins with conflicting results (opposite logFC direction). A total of 119 genes were obtained to be included in the models as restrictions to define mccRCC (Supplementary Table 10).

### Modelling methodology

#### Human protein network, training set and mathematical modelling

The TPMS mathematical models [18] were built over the Human Protein Network (HPN), considering all the proteins from the human proteome and all known protein-protein interactions (PPI) from dedicated databases [18, 20] —including physical interactions and modulations, signalling, metabolic relationships, and gene expression regulation—and manual curation of scientific literature [18]. The models were trained with a collection of known input (drugs)–output (clinical conditions) physiological signals, molecularly defined by literature and database mining; this collection conformed the Training Set that every mathematical model must satisfy [18]. The error was the sum of the input–output relationships that the model did not comply with. Two complementary approaches were employed to evaluate the mechanisms of action (MoAs): Artificial Neural Networks (ANNs), with predictive capacity [88]; and Sampling-based Methods, with descriptive capacity [18].

#### ANN analysis: detecting relationships and additive effects

The predictive power of the mathematical models was exploited through ANN algorithms [89], which attempt to find the shortest distance between protein sets within the HPN and assign a predictive score (ANN value, range 0 to 100%) that quantifies the probability of a functional relationship between the groups of proteins evaluated [88]. Each ANN value is associated with a *p*-value that describes the probability of a true positive result (Table 1).

The relationship between the drugs under study and the mRCC motives was calculated considering their complete drug target profiles and the individual targets. Two parameters were evaluated: the “additive effect” of the combination, and the “positive predictive relationship”. The additive effect of the combined treatment over the individual drugs was predicted when the ANN score for the combination (considering drugs or individual targets) surpassed the ANN score of either drug (or individual target). As previously described [20, 88], the ANN model is trained with drug information (i.e., combined drug target profiles rather than individual targets) and the prediction accuracy is calculated for those drugs with all targets from the human biological network; accordingly, two criteria were applied to identify positive predictive relationships: *p* < 0.05 was set to evaluate the individual drugs or the drug combination; and a less restrictive criterion of *p* < 0.2 was set to evaluate the targets of cabozantinib and PD1, or combination of targets of both drugs. Thus, a positive relationship showing additive effect of the combination was considered either: at drug level, when the combined drugs showed higher score than the individual drugs, with ANN score >78; and at target level, when targets of the combinatory drug showed higher score than targets of individual drugs, and ANN score >47 (medium or higher likeness; Table 1).

#### Sampling methods analyses: description of mechanisms of action

TPMS sampling-based methods [18, 19] generated models like a Multilayer Perceptron of an Artificial Neural Network over the human protein network (HPN). This methodology was used to describe all plausible relationships between an input or stimulus (drug targets) and an output or response (mRCC motives). Particularly, we created two types of models. Firstly, additive models were generated with PD1 inhibitors plus cabozantinib targets, for motives in which an additive role had been previously detected through the ANN methodology, either at the drug or target level. In this case, cabozantinib targets with positive relationship and additive effect were used. Secondly, cabozantinib MoA models were constructed for those motives for which a relationship was predicted to exist between the drug and the corresponding motive (*p*-value <0.05 according to the ANN analysis), and no additive effect was detected with PD1 inhibition, either through ANN, or through additive sampling methods models. Expression data (Supplementary Table 10) [86] was included as restrictions as previously described [18, 24]. Accuracy can be defined as the percentage of compliance of all drug–pathophysiology relationships included in the training set (Supplementary Table 11). Only solutions complying with the training set biological restrictions with a cross-validated accuracy greater than 90% were considered, i.e., only plausible MoAs according to the accepted scientific knowledge.

The resulting proteins subnetwork with non-null outputs and their values will define the drug MoA. “Predicted protein activity” is the value between 1 and −1 that each protein in the MoA subnetwork achieves; to elucidate the impact of both drugs on each pathophysiological motive featuring the condition, we analysed the ability of each treatment to reverse the protein alterations occurring in these pathological mechanisms, according to the molecular characterisation. Thus, we define “reversed proteins” as the proteins that are activated/inactivated by the drug in the opposite activation state than in the mRCC molecular characterisation, with at least |0.1|protein activity. We calculated the percentage of proteins reversed in each motive. These proteins were considered to evaluate the individual and collaborative coverage of the drugs on each additive or cabozantinib MoA models.

## CONCLUSIONS

**Figure 5:**
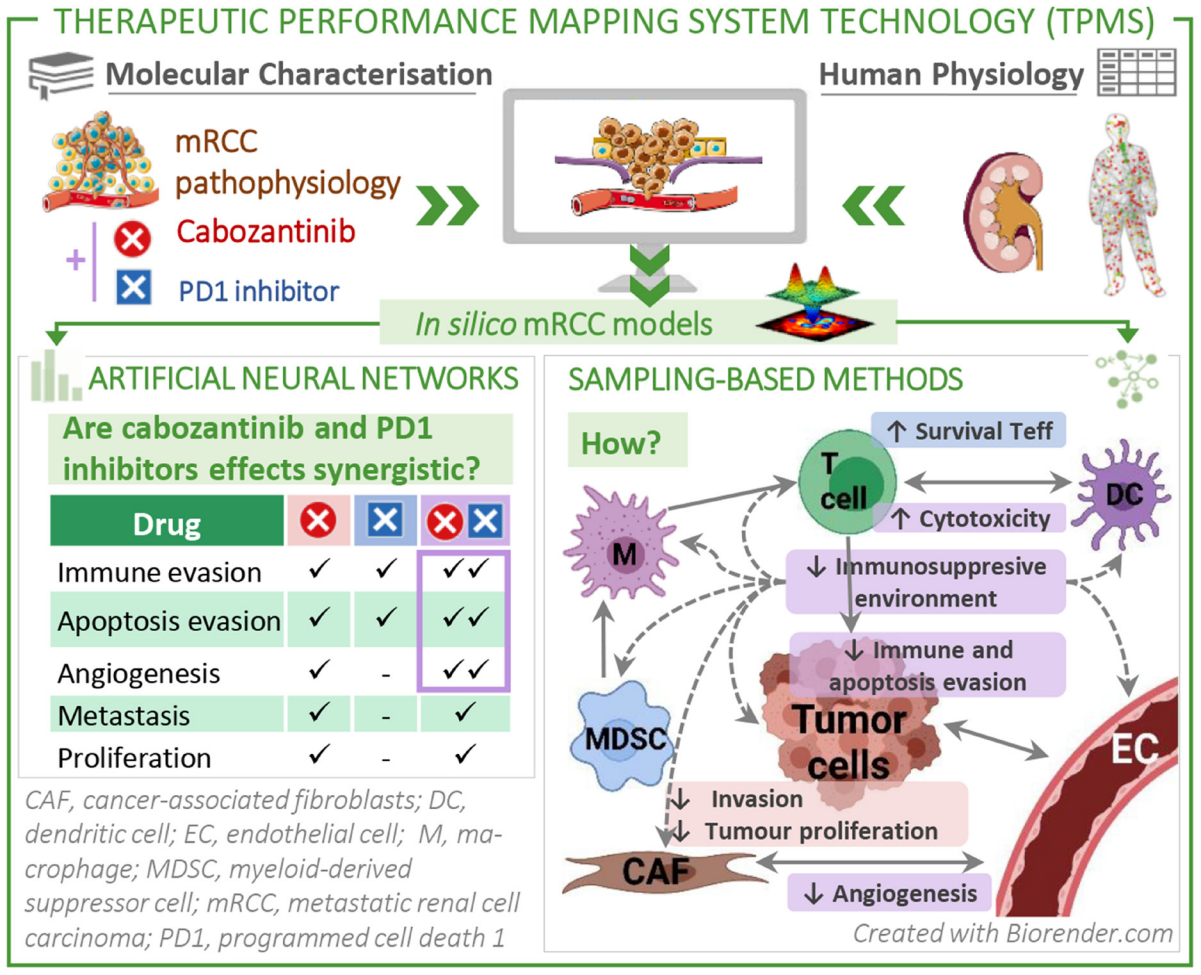
Study overview: We applied systems biology-based machine learning methods to the mRCC environment to find a molecular explanation to the observed synergistic antitumour effect of the combination treatment comprising cabozantinib plus a PD1 inhibitor. The combination therapy creates a therapeutic effect in the tumour and its microenvironment, tackling multiple cellular types and synergizing mainly in the immune evasion domain, and in the angiogenesis and apoptosis hallmarks of cancer to a lesser extent.

The application of novel systems biology– and machine learning–based techniques to the mccRCC environment provides a molecular explanation to the observed synergic antitumour effect of a combo treatment comprising cabozantinib plus a PD1 inhibitor (Figure 5). The combined therapy tackles the full spectrum of mRCC pathophysiology by exerting a wide therapeutical effect both over the tumour cells and the tumour microenvironment that encompasses multiple cellular types and intracellular locations. In our model, cabozantinib was predicted to enhance the known effects of PD1 inhibitors on immune evasion mechanisms through the inhibition of VEGF-VEGFR and Gas6-AXL/TYRO3/MER (TAM) axes modulating multiple humoral and cellular components of the innate and adaptive immune responses. On the other side, PD1 inhibitors were predicted to enhance the antiangiogenic effects of cabozantinib by modulating pro–angiogenic and antiangiogenic factors. Cabozantinib tumour proapoptotic effects are predicted to be also boosted by PD1 inhibition. However, no additive mechanisms were detected to explain these effects, which might be a reflex of T-cell cytotoxicity by PD1 inhibition. In addition, cabozantinib itself was predicted to hamper proliferative signalling and invasive properties and restore cellular adhesion in tumour cells, hallmarks of mesenchymal–epithelial transition. These data based on an AI platform provide a mechanistic rationale and further support for the beneficial combination of cabozantinib and a PD1 inhibitor, and may help guide future nonclinical and clinical research.

## SUPPLEMENTARY MATERIALS




