# Supplementary material for: Exploring the synergistic effects of cabozantinib and a programmed cell death protein 1 inhibitor in metastatic renal cell carcinoma with machine learning
Source: Oncotarget. 2022 Jan 27;13:237–56. doi: 10.18632/oncotarget.28183 (PMC8794707; doi:10.18632/oncotarget.28183)
Supplement: Supplementary file 1 [file oncotarget-13-28183-s001.pdf]

# Exploring the synergistic effects of cabozantinib and a programmed cell death protein 1 inhibitor in metastatic renal cell carcinoma with machine learning

## SUPPLEMENTARY MATERIALS

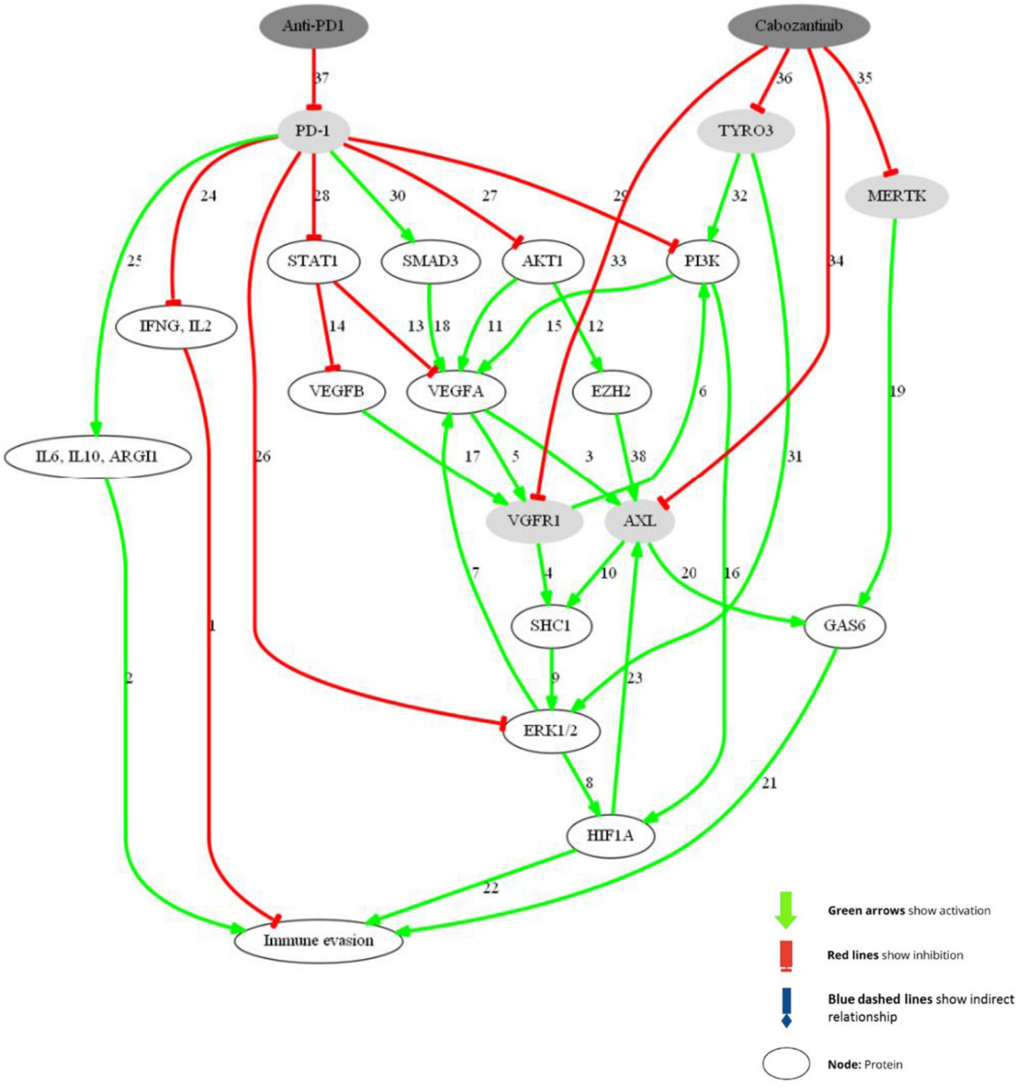


**Supplementary Figure 1: Graphical representation of the predicted MoA of the combination between anti-PD1 agents and cabozantinib in immune evasion processes occurring in mRCC tumours, according to the Sampling Methods model.** Interactions between proteins are detailed in Supplementary Table 7. Interactions are represented as green arrows for activation interactions, red lines for inhibition interactions and blue diamond-ended lines for unspecified interactions (in case they are represented).

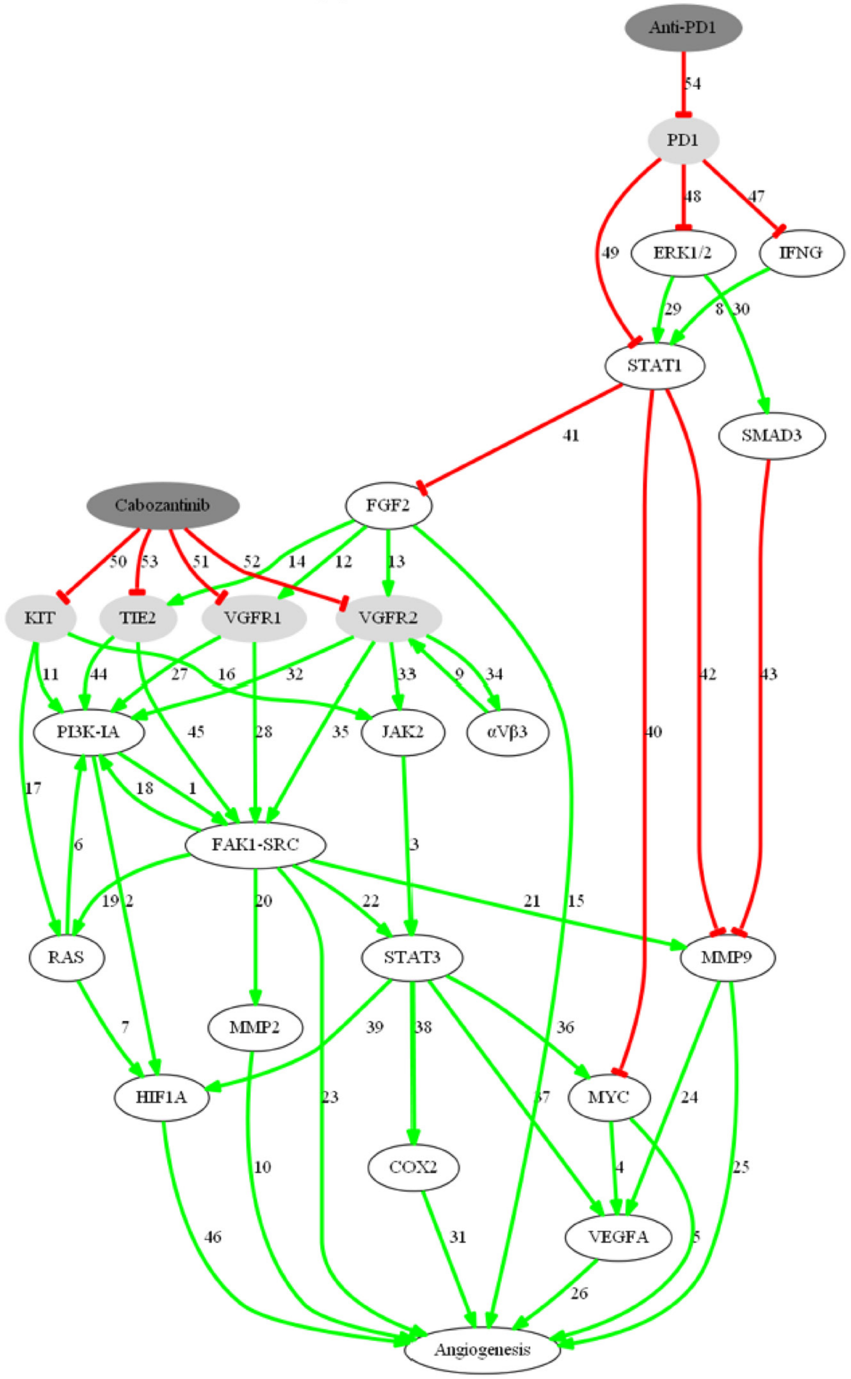


**Supplementary Figure 2: Graphical representation of the predicted MoA of the combination between anti-PD1 agents and cabozantinib in angiogenesis processes occurring in mRCC tumours, according to the sampling methods model.** Interactions between proteins are detailed in Supplementary Table 8. Interactions are represented as green arrows for activation interactions, red lines for inhibition interactions and blue diamond-ended lines for unspecified interactions (in case they are represented).

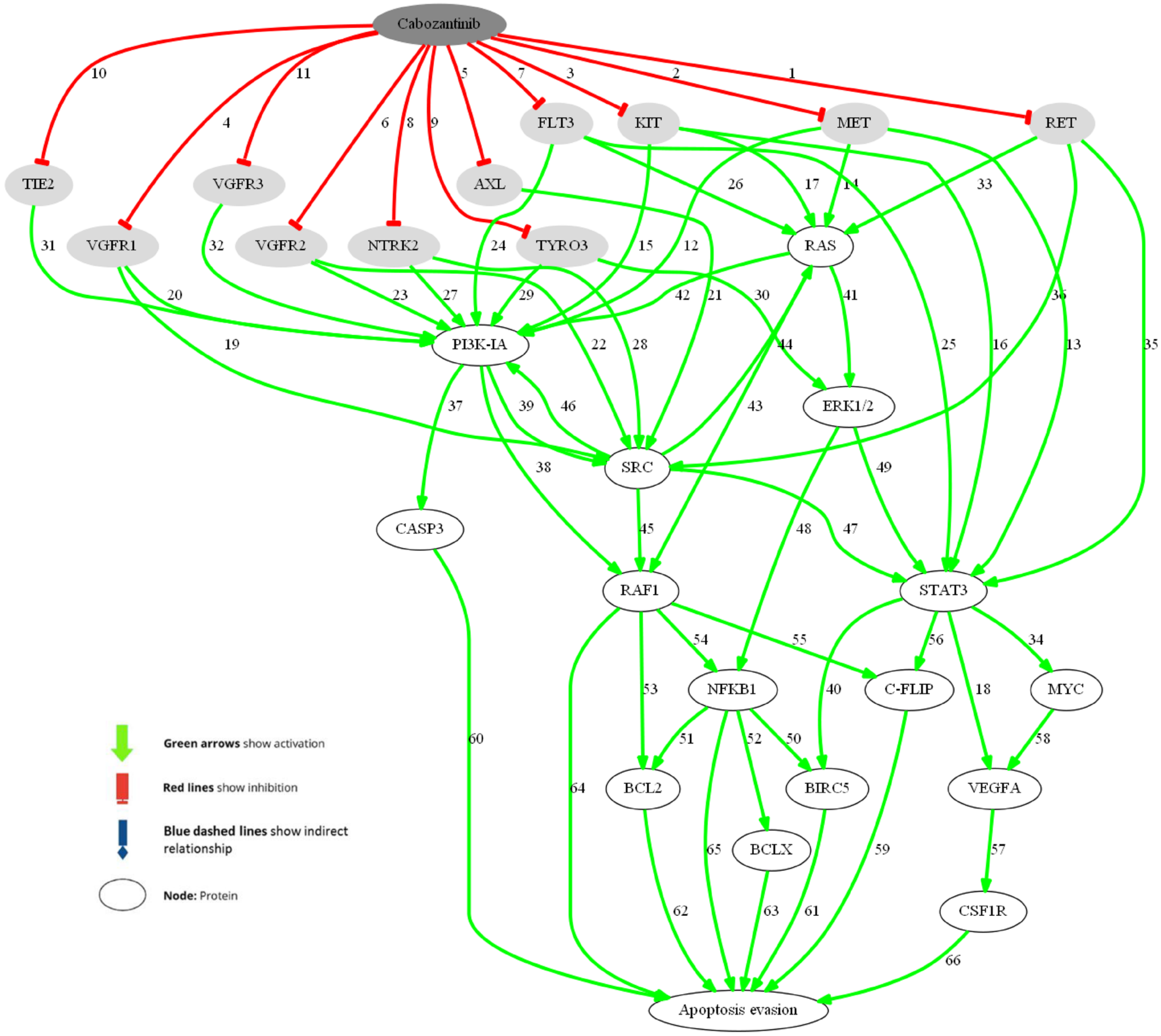


**Supplementary Figure 3: Graphical representation of the predicted MoA of cabozantinib in apoptosis evasion processes occurring in mRCC tumours, according to the sampling methods model.** Interactions between proteins are detailed in Supplementary Table 9. Interactions are represented as green arrows for activation interactions, red lines for inhibition interactions and blue diamond-ended lines for unspecified interactions (in case they are represented).

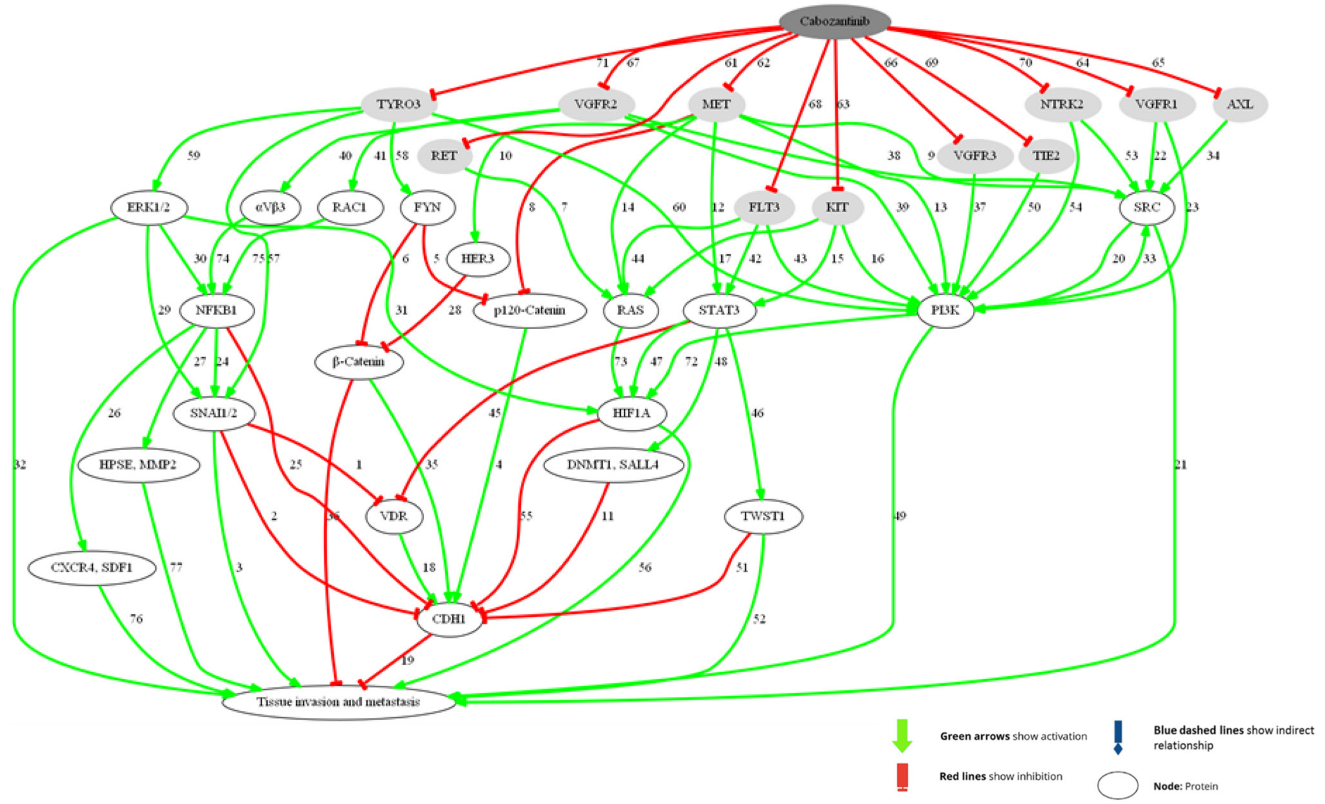


**Supplementary Figure 4: Graphical representation of the predicted MoA of cabozantinib in tissue invasion and metastasis processes occurring in mRCC tumours, according to the sampling methods model.** Interactions between proteins are detailed in Supplementary Table 10. Interactions are represented as green arrows for activation interactions, red lines for inhibition interactions and blue diamond-ended lines for unspecified interactions (in case they are represented).

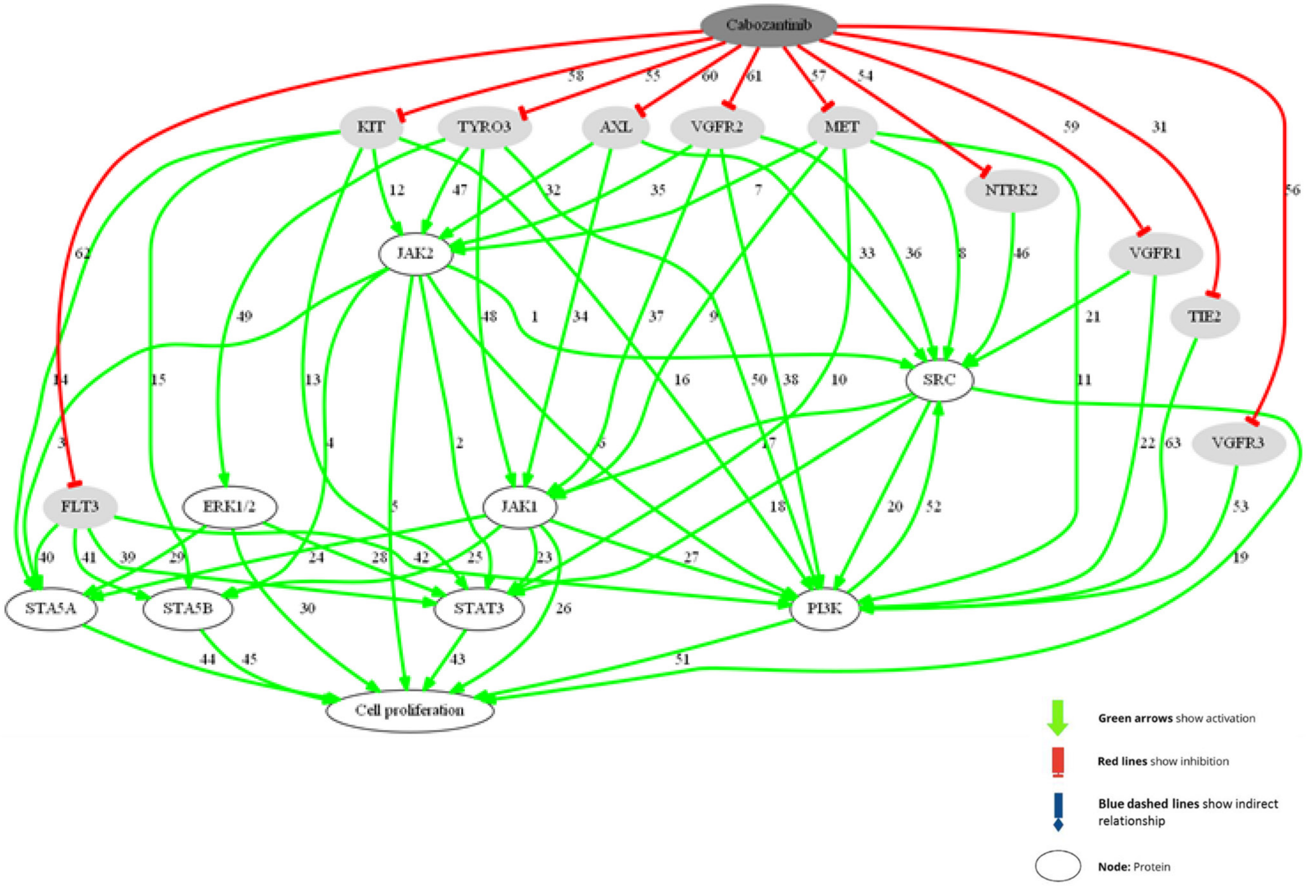


**Supplementary Figure 5: Graphical representation of the predicted MoA of cabozantinib in cell growth and proliferation processes occurring in mRCC tumours, according to the sampling methods model.** Interactions between proteins are detailed in Supplementary Table 11. Interactions are represented as green arrows for activation interactions, red lines for inhibition interactions and blue diamond-ended lines for unspecified interactions (in case they are represented).

**Supplementary Table 1: List of proteins involved in each motive identified in the characterisation phase of metastatic renal cell carcinoma (mRCC) condition.** See Supplementary Table 1

**Supplementary Table 2: Drug targets characterisation.** See Supplementary Table 2

**Supplementary Table 3: mRCC expression data analysis.** See Supplementary Table 3

**Supplementary Table 4: Angiogenesis effector proteins modulated by cabozantinib or anti-PD1 treatments, by means of sampling methods model (see Figure 2)**

| Protein Name | Protein Short Name | Uniprot ID | Altered in PD1 model? | Altered in Cabozantinib model? |
|---|---|---|---|---|
| Myc proto-oncogene protein | MYC | P01106 | ✓ | ✓ |
| Fibroblast growth factor 2 | FGF2 | P09038 | ✓ | ✓ |
| Platelet-derived growth factor receptor beta | PDGFRB | P09619 | ✓ | ✓ |
| Mast/stem cell growth factor receptor Kit | KIT | P10721 | ✓ | ✓ |
| Vascular endothelial growth factor receptor 1 | FLT1 | P17948 | ✓ | ✓ |
| Kit ligand | KITLG | P21583 | ✓ | ✓ |
| Ephrin type-A receptor 2 | EPHA2 | P29317 | ✓ | ✓ |
| Ephrin type-A receptor 3 | EPHA3 | P29320 | ✓ | ✓ |
| Prostaglandin G/H synthase 2 | PTGS2 | P35354 | ✓ | ✓ |
| Vascular endothelial growth factor receptor 2 | KDR | P35968 | ✓ | ✓ |
| Angiopoietin-1 receptor | TEK | Q02763 | ✓ | ✓ |
| Angiopoietin-1 | ANGPT1 | Q15389 | ✓ | ✓ |
| Platelet-derived growth factor subunit B | PDGFB | P01127 | × | ✓ |
| Cathepsin B | CTSB | P07858 | × | ✓ |
| 72 kDa type IV collagenase | MMP2 | P08253 | × | ✓ |
| Proto-oncogene tyrosine-protein kinase Src | SRC | P12931 | × | ✓ |
| Matrix metalloproteinase-9 | MMP9 | P14780 | × | ✓ |
| Vascular endothelial growth factor A | VEGFA | P15692 | × | ✓ |
| Hypoxia-inducible factor 1-alpha | HIF1A | Q16665 | × | ✓ |
| Endothelial PAS domain-containing protein 1 | EPAS1 | Q99814 | × | ✓ |
| Cellular tumor antigen p53 | TP53 | P04637 | ✓ | × |
| Interleukin-12 subunit alpha | IL12A | P29459 | ✓ | × |
| Interleukin-12 subunit beta | IL12B | P29460 | ✓ | × |

The tick marks reflect protein alterations that are reversed by the corresponding treatment, i.e., proteins activated by them when they are inhibited in mRCC, and vice versa (with at least |0.1| protein activity), according to the molecular characterization. The cross indicates absence of a reversion effect.

## Supplementary Table 5: List of specific proteins associated with immune evasion, according to the model

| Link | Protein Name A | UniProt A | Protein Name B | UniProt B | Sign | References |
|---|---|---|---|---|---|---|
| 1 | IL2 | P60568 | Immune evasion | – | −1 | [1] PMID: 14650824 |
| 1 | IFNG | P01579 | Immune evasion | – | −1 | [1] PMID: 25996049; [2] PMID: 14650824 |
| 2 | ARGI1 | P05089 | Immune evasion | – | 1 | [1] PMID: 25996049 |
| 2 | IL6 | P05231 | Immune evasion | – | 1 | [1] PMID: 25996049 |
| 2 | IL10 | P22301 | Immune evasion | – | 1 | [1] PMID: 31013896; [2] PMID: 14650824; [3] PMID: 14650824 |
| 3 | VEGFA | P15692 | AXL | P30530 | 1 | [1] PMID: 22327215 |
| 4 | VGFR1 | P17948 | SHC1 | P29353 | 1 | [1] PMID: 14983012 |
| 5 | VEGFA | P15692 | VGFR1 | P17948 | 1 | [1] PMID: 22866201 |
| 6 | VGFR1 | P17948 | PI3K | Q92569, P27986, O00459, P42336, P42338, O00329 | 1 | [1] PMID: 26693066; [2] PMID: 21212275; [3] PMID: 18284215; [4] PMID: 18079407; [5] PMID: 12814952 |
| 7 | ERK1/2 | P28482; P27361 | VEGFA | P15692 | 1 | [1] PMID: 21904903; [2] PMID: 14737074 |
| 8 | ERK1/2 | P28482; P27361 | HIF1A | Q16665 | 1 | [1] PMID: 27909249; [2] PMID: 10551817 |
| 9 | SHC1 | P29353 | ERK1/2 | P28482, P27361 | 1 | [1] PMID: 17178724 |
| 10 | AXL | P30530 | SHC1 | P29353 | 1 | [1] PMID: 19055724 |
| 11 | AKT1 | P31749 | VEGFA | P15692 | 1 | [1] PMID: 22174934; [2] PMID: 18264090 |
| 12 | AKT1 | P31749 | EZH2 | Q15910 | 1 | [1] PMID: 29556394 |
| 13 | STAT1 | P42224 | VEGFA | P15692 | -1 | [1] PMID: 16585190; [2] PMID: 26631912 |
| 14 | STAT1 | P42224 | VEGFB | P49765 | -1 | [1] PMID: 16585190; [2] PMID: 26631912 |
| 15 | PK3CA | P42336 | VEGFA | P15692 | 1 | [1] PMID: 22144946 |
| 16 | PI3K | Q92569; P27986; O00459; P42336; P42338; O00329 | HIF1A | Q16665 | 1 | [1] PMID: 18160990; [2] PMID: 22144946; [3] PMID: 23590596; [4] PMID: 21922131; [5] PMID: 12874030 |
| 17 | VEGFB | P49765 | VGFR1 | P17948 | 1 | [1] PMID: 19029957 |
| 18 | SMAD3 | P84022 | VEGFA | P15692 | 1 | [1] PMID: 11486006 |
| 19 | MERTK | Q12866 | GAS6 | Q14393 | 1 | [1] PMID: 27028863 |
| 20 | AXL | P30530 | GAS6 | Q14393 | 1 | [1] PMID: 28072762 |
| 21 | GAS6 | Q14393 | Immune evasion | – | 1 | [1] PMID: 25568918 |
| 22 | HIF1A | Q16665 | Immune evasion | – | 1 | [1] PMID: 25563193 |
| 23 | HIF1A | Q16665 | AXL | P30530 | 1 | [1] PMID: 26760782 |
| 24 | PD-1 | Q15116 | IL2 | P60568 | −1 | [1] PMID: 22900886; [2] PMID: 16278812; [3] PMID: 25534622 |
| 24 | PD-1 | Q15116 | IFNG | P01579 | −1 | [1] PMID: 22900886; [2] PMID: 16278812; [3] PMID: 25534622 |
| 25 | PD-1 | Q15116 | IL10 | P22301 | 1 | [1] PMID: 25534622; [2] PMID: 27783986; [3] PMID: 28018338 |
| 25 | PD-1 | Q15116 | IL6 | P05231 | 1 | [1] PMID: 25534622; [2] PMID: 29967259 |
| 25 | PD-1 | Q15116 | ARGI1 | P05089 | 1 | [1] PMID: 27783986 |
| 26 | PD-1 | Q15116 | ERK1/2 | P28482, P27361 | −1 | [1] PMID: 28018338 |
| 27 | PD-1 | Q15116 | AKT1 | P31749 | −1 | [1] PMID: 22740686; [2] PMID: 22900886; [3] PMID: 26297712 |
| 28 | PD-1 | Q15116 | STAT1 | P42224 | −1 | [1] PMID: 22900886 |

| | | | | | | |
|---|---|---|---|---|---|---|
| 29 | PD-1 | Q15116 | PI3K | Q92569, P27986, O00459, P42336, P42338, O00329 | −1 | [1] PMID: 22900886; [2] PMID: 24812408; [3] PMID: 17237390 |
| 30 | PD-1 | Q15116 | SMAD3 | P84022 | 1 | [1] PMID: 28018338; [2] PMID: 25281753 |
| 31 | TYRO3 | Q06418 | ERK1/2 | P28482, P27361 | 1 | [1] PMID: 30501104 |
| 32 | TYRO3 | Q06418 | PI3K | Q92569, P27986, O00459, P42336, P42338, O00329 | 1 | [1] PMID: 30501104 |
| 33 | Cabozantinib | – | VGFR1 | P17948 | −1 | [1] PMID: 21926191 |
| 34 | Cabozantinib | – | AXL | P30530 | −1 | [1] PMID: 21926191 |
| 35 | Cabozantinib | – | MERTK | Q12866 | −1 | [1] PMID: 30679901 |
| 36 | Cabozantinib | – | TYRO3 | Q06418 | −1 | [1] PMID: 29662541 |
| 37 | Anti-PD1 | – | PD-1 | Q15116 | −1 | [1] PMID: 26622321 |
| 38 | EZH2 | Q15910 | AXL | P30530 | 1 | [1] PMID: 23077658 |

The sign next to the UniProt code of each protein indicates the degree of protein activation: (1) activation, (–1) inhibition. The entries for the corresponding articles are listed in the "Reference" column.

## Supplementary Table 6: List of specific proteins associated with angiogenesis, according to the model

| Link | Protein Name A | UniProt A | Protein Name B | UniProt B | Sign | Reference |
|---|---|---|---|---|---|---|
| 1 | PI3K-IA | P42336, P42338, O00329, P27986, O00459, Q92569 | FAK1-SRC | Q05397, P12931 | 1 | [1] PMID: 27169346 |
| 2 | PI3K-IA | P42336, P42338, O00329, P27986, O00459, Q92569 | HIF1A | Q16665 | 1 | [1] PMID: 18160990 |
| 3 | JAK2 | O60674 | STAT3 | P40763 | 1 | [1] PMID: 21333372 |
| 4 | MYC | P01106 | VEGFA | P15692 | 1 | [1] PMID: 24940000 |
| 5 | MYC | P01106 | Angiogenesis | – | 1 | [1] PMID: 24940000 |
| 6 | RAS | P01112, P01116, P01111 | PI3K-IA | P42336, P42338, O00329, P27986, O00459, Q92569 | 1 | [1] PMID: 21779497; [2] PMID: 22683270 |
| 7 | RAS | P01112, P01116, P01111 | HIF1A | Q16665 | 1 | [1] PMID: 23201159 |
| 8 | IFNG | P01579 | STAT1 | P42224 | 1 | [1] PMID: 10637230 |
| 9 | αVβ3 | P06756, P05106 | VGFR2 | P35968 | 1 | [1] PMID: 19267251 |
| 10 | MMP2 | P08253 | Angiogenesis | – | 1 | [1] PMID: 18690841 |
| 11 | KIT | P10721 | PI3K-IA | P42336, P42338, O00329, P27986, O00459, Q92569 | 1 | [1] PMID: 21076081 |
| 12 | FGF2 | P09038 | VGFR1 | P17948 | 1 | [1] PMID: 18625704 |
| 13 | FGF2 | P09038 | VGFR2 | P35968 | 1 | [1] PMID: 32076044 |
| 14 | FGF2 | P09038 | TIE2 | Q02763 | 1 | [1] PMID: 27216150 |
| 15 | FGF2 | P09038 | Angiogenesis | - | 1 | [1] PMID: 28799166; [2] PMID: 16105884 |

| | | | | | | |
|---|---|---|---|---|---|---|
| 16 | KIT | P10721 | JAK2 | O60674 | 1 | [1] PMID: 8611693; [2] PMID: 11494148 |
| 17 | KIT | P10721 | RAS | P01112, P01116, P01111 | 1 | [1] PMID: 20824047 |
| 18 | FAK1-SRC | Q05397, P12931 | PI3K-IA | P42336, P42338, O00329, P27986, O00459, Q92569 | 1 | [1] PMID: 24741093; [2] PMID: 17982280 |
| 19 | FAK1-SRC | Q05397, P12931 | RAS | P01112, P01116, P01111 | 1 | [1] PMID: 25157176; [2] PMID: 25371820; [3] PMID: 22762016 |
| 20 | FAK1-SRC | Q05397, P12931 | MMP2 | P08253 | 1 | [1] PMID: 16453304 |
| 21 | FAK1-SRC | Q05397, P12931 | MMP9 | P14780 | 1 | [1] PMID: 19199380 |
| 22 | FAK1-SRC | Q05397, P12931 | STAT3 | P40763 | 1 | [1] PMID: 12244095 |
| 23 | FAK1-SRC | Q05397, P12931 | Angiogenesis | – | 1 | [1] PMID: 29527128; [2] DOI: 10.1200/JCO.2016.34.15_suppl.e16133 |
| 24 | MMP9 | P14780 | VEGFA | P15692 | 1 | [1] PMID: 14500349 |
| 25 | MMP9 | P14780 | Angiogenesis | – | 1 | [1] PMID: 14500349; [2] PMID: 14530359 |
| 26 | VEGFA | P15692 | Angiogenesis | – | 1 | [1] PMID: 18690841; [2] PMID: 19269025 |
| 27 | VGFR1 | P17948 | PI3K-IA | P42336, P42338, O00329, P27986, O00459, Q92569 | 1 | [1] PMID: 21212275 |
| 28 | VGFR1 | P17948 | FAK1-SRC | Q05397, P12931 | 1 | [1] PMID: 29263797 |
| 29 | ERK1/2 | P27361, P28482 | STAT1 | P42224 | 1 | [1] PMID: 22900886 |
| 30 | ERK1/2 | P27361, P28482 | SMAD3 | P84022 | 1 | [1] PMID: 22880011 |
| 31 | COX2 | P35354 | Angiogenesis | – | 1 | [1] PMID: 15179620 |
| 32 | VGFR2 | P35968 | PI3K-IA | P42336, P42338, O00329, P27986, O00459, Q92569 | 1 | [1] PMID: 17658244 |
| 33 | VGFR2 | P35968 | JAK2 | O60674 | 1 | [1] PMID: 20732905 |
| 34 | VGFR2 | P35968 | αVβ3 | P06756, P05106 | 1 | [1] PMID: 19267251 |
| 35 | VGFR2 | P35968 | FAK1-SRC | Q05397, P12931 | 1 | [1] PMID: 11282421 |
| 36 | STAT3 | P40763 | MYC | P01106 | 1 | [1] PMID: 24928963 |
| 37 | STAT3 | P40763 | VEGFA | P15692 | 1 | [1] PMID: 15735682 |
| 38 | STAT3 | P40763 | COX2 | P35354 | 1 | [1] PMID: 24127267 |
| 39 | STAT3 | P40763 | HIF1A | Q16665 | 1 | [1] PMID: 18985005 |
| 40 | STAT1 | P42224 | MYC | P01106 | −1 | [1] PMID: 10637230 |
| 41 | STAT1 | P42224 | FGF2 | P09038 | −1 | [1] PMID: 11971185 |
| 42 | STAT1 | P42224 | MMP9 | P14780 | −1 | [1] PMID: 19965686 |
| 43 | SMAD3 | P84022 | MMP9 | P14780 | −1 | [1] PMID: 25056536 |
| 44 | TIE2 | Q02763 | PI3K-IA | P42336, P42338, O00329, P27986, O00459, Q92569 | 1 | [1] PMID: 25371820 |
| 45 | TIE2 | Q02763 | FAK1-SRC | Q05397, P12931 | 1 | [1] PMID: 27695111 |
| 46 | HIF1A | Q16665 | Angiogenesis | – | 1 | [1] PMID: 24916472 |
| 47 | PD1 | Q15116 | IFNG | P01579 | −1 | [1] PMID: 22900886 |
| 48 | PD1 | Q15116 | ERK1/2 | P27361, P28482 | −1 | [1] PMID: 28881701 |
| 49 | PD1 | Q15116 | STAT1 | P42224 | −1 | [1] PMID: 22900886 |
| 50 | Cabozantinib | – | KIT | P10721 | −1 | [1] PMID: 21926191 |
| 51 | Cabozantinib | – | VGFR1 | P17948 | −1 | [1] PMID: 21926191 |
| 52 | Cabozantinib | – | VGFR2 | P35968 | −1 | [1] PMID: 21926191 |
| 53 | Cabozantinib | – | TIE2 | Q02763 | −1 | [1] PMID: 21926191 |
| 54 | Anti-PD1 | – | PD1 | Q15116 | −1 | [1] PMID: 26622321 |

The sign next to the UniProt code of each protein indicates the degree of protein activation: (1) activation, (–1) inhibition. The entries for the corresponding articles are listed in the "Reference" column.

**Supplementary Table 7: List of specific proteins associated with apoptosis evasion, according to the model**

| Link | Protein Name A | UniProt A | Protein Name B | UniProt B | Sign | Reference |
|---|---|---|---|---|---|---|
| 1 | C-FLIP | O15519 | Apoptosis evasion | – | 1 | [1] PMID: 12036919; [2] PMID: 29796174; [3] PMID: 23255321 |
| 2 | MYC | P01106 | C-FLIP | O15519 | −1 | [1] PMID: 15367674 |
| 3 | MYC | P01106 | Apoptosis evasion | – | −1 | [1] PMID: 25387056; [2] PMID: 24890832; [3] PMID: 24985130 |
| 4 | RAF1 | P04049 | C-FLIP | O15519 | 1 | [1] DOI: 10.1074/jbc.M113.506428 |
| 5 | RAF1 | P04049 | NFKB1 | P19838 | 1 | [1] PMID: 27899961 |
| 6 | RAF1 | P04049 | Apoptosis evasion | – | 1 | [1] PMID: 16890795; [2] PMID: 18459149 |
| 7 | CSF1R | P07333 | Apoptosis evasion | – | 1 | [1] PMID: 22052465 |
| 8 | RET | P07949 | SRC | P12931 | 1 | [1] PMID: 10070972 |
| 9 | RET | P07949 | PLCG1 | P19174 | 1 | [1] PMID: 23703528; [2] PMID: 22355350 |
| 10 | RET | P07949 | CBL | P22681 | 1 | [1] PMID: 15677445; [2] PMID: 20930041 |
| 11 | RET | P07949 | SHC1 | P29353 | 1 | [1] PMID: 20930041 |
| 12 | RET | P07949 | STAT3 | P40763 | 1 | [1] PMID: 12637586; [2] PMID: 17209045; [3] PMID: 11536047 |
| 13 | RET | P07949 | GRB10 | Q13322 | 1 | [1] PMID: 7665556 |
| 14 | SRC | P12931 | RAF1 | P04049 | 1 | [1] PMID: 15845549 |
| 15 | SRC | P12931 | VEGFA | P15692 | 1 | [1] PMID: 11696015 |
| 16 | SRC | P12931 | STAT3 | P40763 | 1 | [1] PMID: 12244095; [2] PMID: 19372587 |
| 17 | VEGFA | P15692 | CSF1R | P07333 | 1 | [1] PMID: 27629739; [2] PMID: 25249155 |
| 18 | VEGFA | P15692 | Apoptosis evasion | – | 1 | [1] PMID: 10066377; [2] PMID: 21827394 |
| 19 | PLCG1 | P19174 | SRC | P12931 | 1 | [1] PMID: 18594017 |
| 20 | PLCG1 | P19174 | CBL | P22681 | 1 | [1] PMID: 12061819; [2] PMID: 12803489 |
| 21 | NFKB1 | P19838 | C-FLIP | O15519 | 1 | [1] PMID: 11463813 |
| 22 | NFKB1 | P19838 | VEGFA | P15692 | 1 | [1] DOI: 10.1007/s11805-006-0112-2; [2] PMID: 20127012 |
| 23 | NFKB1 | P19838 | Apoptosis evasion | – | 1 | [1] PMID: 31462894; [2] PMID: 25936818 |
| 24 | NFKB1 | P19838 | Survivin | O15392 | 1 | [1] PMID: 15880597 |
| 24 | NFKB1 | P19838 | PGH2 | P35354 | 1 | [1] PMID: 18286508; [2] PMID:19321801 |
| 25 | CBL | P22681 | MYC | P01106 | −1 | [1] PMID: 18413713 |
| 26 | PGH2 | P35354 | Apoptosis evasion | – | 1 | [1] PMID: 31462894; [2] PMID: 20339581 |
| 26 | Survivin | O15392 | Apoptosis evasion | – | 1 | [1] PMID: 31462894; [2] PMID: 29604934; [3] PMID: 29301217; [4] PMID: 12036919 |
| 27 | CBL | P22681 | SRC | P12931 | 1 | [1] PMID: 11149930 |
| 28 | SHC1 | P29353 | CBL | P22681 | 1 | [1] PMID: 15677445; [2] PMID: 20930041 |
| 29 | STAT3 | P40763 | C-FLIP | O15519 | 1 | [1] PMID: 30158588 |
| 30 | STAT3 | P40763 | VEGFA | P15692 | 1 | [1] PMID: 15735682; [2] PMID: 19185840; [3] PMID: 11960372 |
| 31 | STAT3 | P40763 | NFKB1 | P19838 | 1 | [1] PMID: 12057007; [2] PMID: 21187858; [3] PMID: 23915189; [4] PMID: 21364020 |
| 32 | STAT3 | P40763 | Survivin | O15392 | 1 | [1] PMID: 16397018; [2] PMID: 15077160; [3] PMID: 12036919 |
| 32 | STAT3 | P40763 | PGH2 | P35354 | 1 | [1] PMID: 24127267 |
| 33 | GRB10 | Q13322 | RAF1 | P04049 | 1 | [1] PMID: 9553107 |
| 34 | Cabozantinib | - | RET | P07949 | −1 | [1] PMID: 30679901 |

The sign next to the UniProt code of each protein indicates the degree of protein activation: (1) activation, (−1) inhibition. The entries for the corresponding articles are listed in the "Reference" column.

**Supplementary Table 8: List of specific proteins associated with tissue invasion and metastasis, according to the model.** See Supplementary Table 8

**Supplementary Table 9: List of specific proteins associated with cell growth and proliferation, according to the model**

| Link | Protein Name A | UniProt A | Protein Name B | UniProt B | Sign | Reference |
|---|---|---|---|---|---|---|
| 1 | JAK2 | O60674 | SRC | P12931 | 1 | [1] KEGG: map04917 Prolactin signaling pathway; [2] PMID: 21442038 |
| 2 | JAK2 | O60674 | STAT3 | P40763 | 1 | [1] PMID: 25342631; [2] PMID: 9396763; [3] PMID: 29039526; [4] PMID: 26750311; [5] PMID:109252978; [6] PMID:15284024 |
| 3 | JAK2 | O60674 | STA5A | P42229 | 1 | [1] PMID: 22125600; [2] PMID: 9047382; [3] PMID: 9575217; [4] map04630 Jak-STAT signaling pathway; [5] PMID: 9396763; [6] PMID: 9575217 |
| 4 | JAK2 | O60674 | STA5B | P51692 | 1 | [1] PMID: 24470975; [2] PMID: 9047382; [3] KEGG: map04630 Jak-STAT signaling pathway; [4] PMID: 9396763 |
| 5 | JAK2 | O60674 | Cell growth and proliferation | – | 1 | [1] PMID: 17898043 |
| 6 | JAK2 | O60674 | PI3K (PK3CA, PK3CB, P85A) | P42336, P42338, P27986 | 1 | [1] KEGG: hsa04151 PI3K-Akt signaling pathway; [2] PMID: 24583800; [3] DOI: 10.1016/j.ccr.2012.10.023; [4] PMID: 28495456; |
| 7 | MET | P08581 | JAK2 | O60674 | 1 | [1] PMID: 30158543; [2] PMID: 29735799 |
| 8 | MET | P08581 | SRC | P12931 | 1 | [1] PMID: 28810597 |
| 9 | MET | P08581 | JAK1 | P23458 | 1 | [1] PMID: 24931611; [2] PMID: 30723303 |
| 10 | MET | P08581 | STAT3 | P40763 | 1 | [1] PMID: 25801713; [2] PMID: 22128289; [3] PMID: 12168776 |
| 11 | MET | P08581 | PI3K (PK3CA, PK3CB, P85A) | P42336, P42338, P27986 | 1 | [1] PMID: 12611639; [2] PMID: 20584748; [3] PMID: 23576023 |
| 12 | KIT | P10721 | JAK2 | O60674 | 1 | [1] PMID: 23058498; [2] PMID: 8611693 |
| 13 | KIT | P10721 | STAT3 | P40763 | 1 | [1] PMID: 28042518; [2] PMID: 11494148 |
| 14 | KIT | P10721 | STA5A | P42229 | 1 | [1] PMID: 10358045 |
| 15 | KIT | P10721 | STA5B | P51692 | 1 | [1] PMID: 10358045 |
| 16 | KIT | P10721 | PI3K (PK3CA, PK3CB, P85A) | P42336, P42338, P27986 | 1 | [1] PMID: 21076081; [2] PMID: 26474484 |
| 17 | SRC | P12931 | JAK1 | P23458 | 1 | [1] PMID: 16393697 |
| 18 | SRC | P12931 | STAT3 | P40763 | 1 | [1] PMID: 12244095; [2] PMID: 19372587 |
| 19 | SRC | P12931 | Cell growth and proliferation | – | 1 | [1] PMID: 29527128; [2] DOI: 10.1200/ JCO.2016.34.15_suppl.e16133 |
| 20 | SRC | P12931 | PI3K (PK3CA, PK3CB, P85A) | P42336, P42338, P27986 | 1 | [1] KEGG: hsa04370-VEGF signaling pathway; [2] PMID: 24741093; [3] PMID: 29487290; [4] PMID: 12933816 |
| 21 | VGFR1 | P17948 | SRC | P12931 | 1 | [1] PMID: 29263797 |
| 22 | VGFR1 | P17948 | PI3K (PK3CA, PK3CB, P85A) | P42336, P42338, P27986 | 1 | [1] PMID: 12814952; [2] PMID: 18079407; [3] PMID: 26693066 |
| 23 | JAK1 | P23458 | STAT3 | P40763 | 1 | [1] PMID: 11163768; [2] KEGG: hsa04630-Jak-STAT signaling pathway; [3] PMID: 17908914 |
| 24 | JAK1 | P23458 | STA5A | P42229 | 1 | [1] PMID: 9047382 |
| 25 | JAK1 | P23458 | STA5B | P51692 | 1 | [1] PMID: 9047382 |
| 26 | JAK1 | P23458 | Cell growth and proliferation | – | 1 | [1] PMID: 17898043 |
| 27 | JAK1 | P23458 | PI3K (PK3CA, PK3CB, P85A) | P42336, P42338, P27986 | 1 | [1] PMID: 17982039; [2] PMID: 22711705 |
| 28 | ERK1/2 | P28482, P27361 | STAT3 | P40763 | 1 | [1] PMID: 11350938; [2] PMID: 12763138 |
| 29 | ERK1/2 | P28482, P27361 | STA5A | P42229 | 1 | [1] PMID: 10194762 |

| | | | | | | |
|---|---|---|---|---|---|---|
| 30 | ERK1/2 | P28482, P27361 | Cell growth and proliferation | – | 1 | [1] PMID: 18172299; [2] PMID: 26732239; [3] PMID: 31477752 |
| 31 | Cabozantinib | – | 1 | Q02763 | –1 | [1] PMID: 21926191 |
| 32 | AXL | P30530 | JAK2 | O60674 | 1 | [1] PMID: 24186067 |
| 33 | AXL | P30530 | SRC | P12931 | 1 | [1] PMID: 25187556 |
| 34 | AXL | P30530 | JAK1 | P23458 | 1 | [1] DOI: 10.1158/0008-5472.CAN-19-3183 |
| 35 | VGFR2 | P35968 | JAK2 | O60674 | 1 | [1] PMID: 20732905; [2] PMID: 25151964 |
| 36 | VGFR2 | P35968 | SRC | P12931 | 1 | [1] PMID: 11282421; [2] PMID: 22689825 |
| 37 | VGFR2 | P35968 | JAK1 | P23458 | 1 | [1] PMID: 26981109 |
| 38 | VGFR2 | P35968 | PI3K (PK3CA, PK3CB, P85A) | P42336, P42338, P27986 | 1 | [1] PMID: 17658244; [2] KEGG: hsa04370 - VEGF signaling pathway; [3] PMID: 29527128 |
| 39 | FLT3 | P36888 | STAT3 | P40763 | 1 | [1] PMID: 24639346 |
| 40 | FLT3 | P36888 | STA5A | P42229 | 1 | [1] PMID: 14504097; [2] PMID: 12796379 |
| 41 | FLT3 | P36888 | STA5B | P51692 | 1 | [1] PMID: 14504097; [2] PMID: 12796379 |
| 42 | FLT3 | P36888 | PI3K (PK3CA, PK3CB, P85A) | P42336, P42338, P27986 | 1 | [1] KEGG: 05200-Pathways in cancer; [2] PMID: 12951584; [3] PMID: 15840695 |
| 43 | STAT3 | P40763 | Cell growth and proliferation | – | 1 | [1] PMID: 17898043 |
| 44 | STA5A | P42229 | Cell growth and proliferation | – | 1 | [1] PMID: 17898043 |
| 45 | STA5B | P51692 | Cell growth and proliferation | – | 1 | [1] PMID: 17898043 |
| 46 | NTRK2 | Q16620 | SRC | P12931 | 1 | [1] PMID: 19927149 |
| 47 | TYRO3 | Q06418 | JAK2 | O60674 | 1 | [1] PMID: 30501104 |
| 48 | TYRO3 | Q06418 | JAK1 | P23458 | 1 | [1] PMID: 30501104 |
| 49 | TYRO3 | Q06418 | ERK1/2 | P28482, P27361 | 1 | [1] PMID: 30501104 |
| 50 | TYRO3 | Q06418 | PI3K (PK3CA, PK3CB, P85A) | P42336, P42338, P27986 | 1 | [1] PMID: 30501104 |
| 51 | PI3K (PK3CA, PK3CB, P85A) | P42336, P42338, P27986 | Cell growth and proliferation | – | 1 | [1] PMID: 25387056; [2] PMID: 25924008 |
| 52 | PI3K (PK3CA, PK3CB, P85A) | P42336, P42338, P27986 | SRC | P12931 | 1 | [1] PMID: 27169346 |
| 53 | VGFR3 | P35916 | PI3K (PK3CA, PK3CB, P85A) | P42336, P42338, P27986 | 1 | [1] PMID: 22745786; [2] PMID: 22745786; [3] PMID: 20814228 |
| 54 | Cabozantinib | – | NTRK2 | Q16620 | –1 | [1] Cometriq: EPAR - Product Information. 2014, European Medicines Agency |
| 55 | Cabozantinib | – | TYRO3 | Q06418 | –1 | [1] PMID: 29662541 |
| 56 | Cabozantinib | – | VGFR3 | P35916 | –1 | [1] PMID: 21613405 |
| 57 | Cabozantinib | – | MET | P08581 | –1 | [1] PMID: 21926191 |
| 58 | Cabozantinib | – | KIT | P10721 | –1 | [1] PMID: 21926191 |
| 59 | Cabozantinib | – | VGFR1 | P17948 | –1 | [1] PMID: 21926191 |
| 60 | Cabozantinib | – | AXL | P30530 | –1 | [1] PMID: 21926191 |
| 61 | Cabozantinib | – | VGFR2 | P35968 | –1 | [1] PMID: 21926191 |
| 62 | Cabozantinib | – | FLT3 | P36888 | –1 | [1] PMID: 21926191 |
| 63 | TIE2 | Q02763 | PI3K (PK3CA, PK3CB, P85A) | P42336, P42338, P27986 | 1 | [1] PMID: 21130043; [2] PMID: 25371820 |

The sign next to the UniProt code of each protein indicates the degree of protein activation: (1) activation, (–1) inhibition. The entries for the corresponding articles are listed in the "Reference" column.

**Supplementary Table 10: Immune evasion effector proteins modulated by cabozantinib or anti-PD1 treatments, by means of Sampling Methods model (see Figure 2)**

| Protein Name | Protein Short Name | Uniprot ID | Altered in PD1 model? | Altered in Cabozantinib model? |
|---|---|---|---|---|
| Interferon gamma | IFNG | P01579 | ✓ | ✓ |
| Interleukin-8 | CXCL8 | P10145 | ✓ | ✓ |
| Vascular endothelial growth factor receptor 1 | FLT1 | P17948 | ✓ | ✓ |
| Interleukin-10 | IL10 | P22301 | ✓ | ✓ |
| Interleukin-12 subunit alpha | IL12A | P29459 | ✓ | ✓ |
| Interleukin-12 subunit beta | IL12B | P29460 | ✓ | ✓ |
| C-X-C motif chemokine 5 | CXCL5 | P42830 | ✓ | ✓ |
| CD83 antigen | CD83 | Q01151 | ✓ | ✓ |
| Transforming growth factor beta-1 proprotein | TGFB1 | P01137 | × | ✓ |
| Granulocyte-macrophage colony-stimulating factor | CSF2 | P04141 | × | ✓ |
| Granzyme B | GZMB | P10144 | × | ✓ |
| HLA class I histocompatibility antigen, alpha chain G | HLA-G | P17693 | × | ✓ |
| Tumor necrosis factor receptor superfamily member 1A | TNFRSF1A | P19438 | × | ✓ |
| Tyrosine-protein kinase receptor UFO | AXL | P30530 | × | ✓ |
| Nitric oxide synthase, inducible | NOS2 | P35228 | × | ✓ |
| Tyrosine-protein kinase receptor TYRO3 | TYRO3 | Q06418 | × | ✓ |
| Tyrosine-protein kinase Mer | MERTK | Q12866 | × | ✓ |
| Growth arrest-specific protein 6 | GAS6 | Q14393 | × | ✓ |
| Caspase-8 | CASP8 | Q14790 | × | ✓ |
| Hypoxia-inducible factor 1-alpha | HIF1A | Q16665 | × | ✓ |
| Forkhead box protein O3 | FOXO3 | O43524 | ✓ | × |
| Arginase-1 | ARG1 | P05089 | ✓ | × |
| Interleukin-6 | IL6 | P05231 | ✓ | × |
| Protein S100-A9 | S100A9 | P06702 | ✓ | × |
| Indoleamine 2,3-dioxygenase 1 | IDO1 | P14902 | ✓ | × |
| Nuclear factor NF-kappa-B p105 subunit | NFKB1 | P19838 | ✓ | × |
| Tumor necrosis factor receptor superfamily member 5 | CD40 | P25942 | ✓ | × |
| Interleukin-15 | IL15 | P40933 | ✓ | × |
| Interleukin-2 | IL2 | P60568 | ✓ | × |
| Nuclear factor NF-kappa-B p100 subunit | NFKB2 | Q00653 | ✓ | × |
| Programmed cell death protein 1 | PDCD1 | Q15116 | ✓ | × |

The tick marks reflect protein alterations that are reversed by the corresponding treatment, i.e., proteins activated by them when they are inhibited in mRCC, and vice versa (with at least |0.1| protein activity), according to the molecular characterization. The cross indicates absence of a reversion effect.

**Supplementary Table 11: Data sources for generation and training of the mathematical models and balance of molecular restrictions**

| Data type | Number of entries |
|---|---|
| **In-house databases information** | |
| Considered Interactions | 437.034 |
| Considered Proteins | 16.918 |
| Characterized Drugs | 5.414 |
| Drug Targets | 2.690 |
| Characterized Clinical Conditions | 253 |
| Clinical Conditions Key Proteins Characterized | 4.076 |
| **Training set information** | |
| Curated drug-indications restrictions | 180,264 (1,731 positive) |
| Drug-ADRs restrictions | 30,096 (2,640 positive) |
| Drug-indications/ADRs protein correlations | 2.655 |

In-house databases information comprise summarised data (number of entries in the database for each data type) used for model construction (network and training set). The training set information indicates the type and quantity of molecular restrictions used to generate the mathematical models. Abbreviation: ADR: Adverse Drug Reactions.
